# Hypoxic BMSC-derived exosomal miRNAs promote metastasis of lung cancer cells via STAT3-induced EMT

**DOI:** 10.1186/s12943-019-0959-5

**Published:** 2019-03-13

**Authors:** Xina Zhang, Buqing Sai, Fan Wang, Lujuan Wang, Yuhui Wang, Leliang Zheng, Guiyuan Li, Jingqun Tang, Juanjuan Xiang

**Affiliations:** 1 Hunan Cancer Hospital, The Affiliated Cancer Hospital of Xiangya School of Medicine, Central South University, Changsha, Hunan China; 2 The Key Laboratory of Carcinogenesis of the Chinese Ministry of Health, Cancer Research Institute, School of Basic Medical Science, Central South University, Changsha, Hunan China; 3 Department of Thoracic Surgery, the Second Xiangya Hospital, Central South University, Changsha, 410013 Hunan China; 4 The Key Laboratory of Carcinogenesis and Cancer Invasion of the Chinese Ministry of Education, Xiangya Hospital, Central South University, Changsha, Hunan China; 5 Hunan Key Laboratory of Nonresolving Inflammation and Cancer, Changsha, 410013 Hunan China

**Keywords:** BMSCs, exosomal miRNA, Metastasis, EMT, STAT3

## Abstract

**Background:**

Metastasis is the main cause of lung cancer mortality. Bone marrow-derived mesenchymal stem cells (BMSCs) are a component of the cancer microenvironment and contribute to cancer progression. Intratumoral hypoxia affects both cancer and stromal cells. Exosomes are recognized as mediators of intercellular communication. Here, we aim to further elucidate the communication between BMSC-derived exosomes and cancer cells in the hypoxic niche.

**Methods:**

Exosomal miRNA profiling was performed using a microRNA array. Lung cancer cells and an in vivo mouse syngeneic tumor model were used to evaluate the effects of select exosomal microRNAs. Hypoxic BMSC-derived plasma exosomal miRNAs were assessed for their capacity to discriminate between cancer patients and non-cancerous controls and between cancer patients with or without metastasis.

**Results:**

We demonstrate that exosomes derived from hypoxic BMSCs are taken by neighboring cancer cells and promote cancer cell invasion and EMT. Exosome-mediated transfer of select microRNAs, including miR-193a-3p, miR-210-3p and miR-5100, from BMSCs to epithelial cancer cells activates STAT3 signaling and increases the expression of mesenchymal related molecules. The diagnostic accuracy of individual microRNA showed that plasma exosomal miR-193a-3p can discriminate cancer patients from non-cancerous controls. A panel of these three plasma exosomal microRNAs showed a better diagnostic accuracy to discriminate lung cancer patients with or without metastasis than individual exosomal microRNA.

**Conclusions:**

Exosome-mediated transfer of miR-193a-3p, miR-210-3p and miR-5100, could promote invasion of lung cancer cells by activating STAT3 signalling-induced EMT. These exosomal miRNAs may be promising noninvasive biomarkers for cancer progression.

**Electronic supplementary material:**

The online version of this article (10.1186/s12943-019-0959-5) contains supplementary material, which is available to authorized users.

## Background

Lung cancer is the leading cause of cancer-related mortality. Exosomes are extracellular membrane vesicles that contain proteins and RNAs. The molecular composition of exosomes is stable and tissue-specific, and can reflect the conditional state of their tissue of origin [1]. mRNAs and microRNAs (miRNAs) have been identified in exosomes [2]. miRNAs are 19-22 nt non-coding RNAs. Circulating miRNAs are protected from degradation by sequestration in exosomes [3]. Increased levels of specific miRNAs assembled on the exosomes have been associated with cancer progression [4–6]. Exosomes are the major mediators of cell–cell communication because they can be captured by neighboring cells [7]. Exosome-mediated transfer of particular miRNAs from stromal cells to epithelial cancer cells contributes to cancer progression [8]. Exosomes are excreted by many cells and can be detected in bodily fluids including blood, urine and bronchoalveolar secretions, which are considered to ideal source of biomarkers [9–11]. There is significant interest in roles of exosomal miRNAs in the development of cancer, especially in the non-invasive detection of cancer progression.

Tumor cells and microenvironmental cells are subjected to a range of stressors such as hypoxia, metabolic stress, starvation, oxidative stress [12]. Hypoxia is a feature of solid tumours [13].The rapid growth of cancer cells exceeds oxygen delivery, producing local hypoxia. Most solid tumours have regions permanently or transiently subjected to hypoxia [14].Hypoxia is characterized by an oxygen tension below physiological normoxia. Hypoxia enhances the release of exosomes containing CD63 and CD9 [15]. Stress can alter the content of exosomes [12].

Bone marrow-derived mesenchymal stem cells (BMSCs) are multipotent stromal cells that are recruited to tumours and contribute to cancer progression. Although bone marrow is considered a tissue with limited oxygen supply [16], the oxygen tension in the centre of solid tumours tissue even lower-close to 0.025 kPa (0.1–0.2%). The microenvironment encountered by BMSCs differs between bone marrow and the cancer microenvironment. In this study, we aimed to elucidate the cell-cell communication in the hypoxic cancer microenvironment mediated by BMSC-derived exosomal miRNAs.

## Methods

### Patients and specimens

Patients diagnosed with lung cancer (*n* = 41), liver cancer (*n* = 21) and pancreatic cancer (*n* = 10) were included in this study. Thirty healthy control were enrolled as a control group. All cases enrolled in this study were identified and diagnosed between December 2017 and May 2018 at the Second Xiangya hospital, Central South University, China. The clinical characteristics of patients are listed in Table 1. Human bone marrow mesenchymal stem cells were obtained from bone marrow aspirates of non-hematological malignant tumor patients. Human bone marrow aspirates and plasma sample were collected from the Second Xiangya Hospital, Central South University. The collection of bone marrow and blood was performed for diagnostic purposes. All human experiments were performed in accordance with the Declaration of Helsinki. Collections and use of these samples were approved by the ethical review committees of the Second Xiangya Hospital. The patients were informed about the sample collection and have signed informed consent forms.

**Table 1:** The clinical characteristics of patients

Clinicopathological features	n	metastatic	non-metastatic	*P* value
Gender				
Male	51	19	32	
Female	21	6	15	0.4887
Age				
≥ 50	40	16	24	
< 50	32	9	23	0.2996
Chemotherapy or radiation				
Yes	30	10	20	
No	42	15	27	0.8371
Tumor cancer				
Lung cancer	41	21	20	
Liver cancer	21	1	20	
Pancreatic cancer	10	3	7	

### Reagent, cell culture and hypoxia treatment

STAT3 inhibitor stattics was purchased from Selleck Chemicals (S7024, Houston, TX, USA). GW4869, an inhibitor of exosomes biogenesis/release, was purchased form Sigma (D1692, St. Louis, MO, USA) .miR-193-3p mimics, miR-210-3p mimics, miR-5100 mimics and corresponding inhibitors were designed and synthesized by RIBOBIO (Gongzhou, China).NSCLC cell lines including H358, A549, H460 were cultured in RPMI-1640 medium(SH30809.01B, Hyclone) and murine lewis lung cancinoma(LLC) cells were cultured in DMEM high glucose medium(SH30022.01B, Hyclone) supplemented with penicillin G (100 U/mL) and streptomycin (100 mg/mL) (SV30010, Hyclone) and 10% fetal bovine serum (04–001-1ACS, BI). Cells were obtained from Cell Bank of Cancer Research Institute, Central South University and authenticated by STR profiling. Cells were routinely sub-cultured using 0.25% (*w*/*v*) trypsin-EDTA solution(SV30042.01, Hyclone). Murine bone marrow-derived MSCs (mBMSCs) from C57BL/6 mice were purchased from Cyagen (Guangzhou, China). mBMSCs were cultured in MEM medium (SH30024.01, Hyclone) supplemented with penicillin G (100 U/mL) and streptomycin (100 mg/mL) (SV30010, Hyclone)and 10% fetal calf serum (04–001-1 ACS, BI).Human MSCs were obtained from bone marrow aspirates of non-hematological malignant tumor patients and were isolated as described previously [17, 18]. Human bone marrow-derived mesenchymal stem cells (hBMSCs) are cultured in Medium For Mesenchymal Stem Cells (HUXMA-03011-440,cyagen). Cells were grown at 37 °C in a humidified atmosphere with 5% CO2. For hypoxia induction, cells were incubated for 3 days in 0.2% oxygen concentration, 37 °C temperature, 5% CO2 concentration and 90% humidity in Hypoxic Workstation (Don Whitley,UK). The hypoxia treated medium was collected for the following experiments. Cells were lysed for extraction of protein and RNA in the workstation to avoid reoxygenation.

### Exosome isolation and quantification

Before exosome isolation, BMSCs were cultured in medium supplemented with exosome-depleted FBS to avoid interference from bovine exosomes [19, 20]. FBS was depleted of exosomes by ultracentrifugation at 1 × 10^5^ g at 4 °C for 16 h (Beckman Coulter Avanti J-30I, USA), then the supernatant of FBS was collected and filtered with a 0.22um filter (millipore,USA). mBMSCs and hBMSCs were cultured in medium with 10% exosome-free FBS. After 72 h, cell culture medium was collected, and exosomes were isolated from the supernatant by differential centrifugation of 300 g for 10 min, 2000 g for 15 min, and 12,000 g for 30 min to remove floating cells and cellular debris. The supernatant was then passed through a 0.22-μm filter(millipore, USA). The resultant supernatant was forced through the membrane by centrifugal filtration at 4 × 10^3^ g for 1 h at 4 °C with the ultrafiltration device (UFC900396,Millipore,USA). Finally, the EXO Quick-TC™ Exosome Isolation Reagent(EXOTC50A-1,System Biosciences,USA) was added in the concentrated solution of supernatant at a ratio of 1:5. Subsequent exosome extraction was conducted according to the kit instructions (SBI, System Biosciences) [21, 22].

Exosomes were isolated from plasma of cancer patients and healthy control according to the manufacturer’s instructions (SBI, System Biosciences) [21]. Briefly, blood was collected into EDTA - K2 anticoagulant tube and mixed immediately to avoid clotting. After centrifuged at 3000 g for 15 min at 4 °C, the supernatant was collected. A total of 250 uL of plasma was mixed with ExoQuick exosome precipitation solution and exosome isolation was performed according to the manufacturer’s instructions (SBI, System Biosciences) [21]. Exosomal protein was measured by the BCA™ Protein Assay Kit (Pierce, USA) [23]. CD63 and HSP70 (ExoAb Antibody Kit,EXOAB-KIT-1, System Biosciences,USA), β-actin (20536-1-AP, Proteintech, Chicago, USA) were used as positive control and inner control for exosomes. The exosomes were used for following experiments immediately or stored at − 80 degrees.

### Transmission electron microscopy (TEM)

The exosomes were observed by TEM [20]. Exosomes were isolated and fixed in 1% glutaraldehyde for 10 min, washed with deionized water. Approximately 10 μL of exosome suspension was placed on formvar carbon-coated 300-mesh copper electron microscopy grids(Agar Scientific Ltd., Stansted,UK), and incubated for 5 min at room temperature. Then exosomes were negatively stained with 2% uranyl oxalate for 1 min at room temperature. The grids were washed with three times of PBS and air dried for 5 min. Images were obtained by TEM (JEM-2100, Jeol, Japan).

### Nanoparticle tracking analysis

The size distribution and concentration of exosomes were determined by Nanoparticle Tracking Analysis (NTA) according to the manufacturer’s instruction, which utilizes the properties of both light scattering and Brownian motion (Zetasizer Nano ZS90 instrument, Malvern, UK). Exosomes were resuspended in 1 ml PBS and mixed, then the diluted exosomes were injected into the Zetasizer Nano ZS90 instrument (Malvern, UK).Particles were tracked and size of particles was measured based on Brownian motion and the diffusion coefficient. Filtered PBS was used as controls. All samples were measured with parameters of 44.5 mm and 0.64 V voltage using NP100 membranes. Samples were calibrated by CPC100 standard particles diluted 1000-fold under identical settings [24]. Five videos of typically 60 s’ duration were taken. Data was analyzed by Zetasizer software (Malvern Instruments) which was optimized to identify and track each particle on a frame-by-frame basis.

### RNA exaction from cells and exosomes

Isolation of cellular or extracellular RNA was performed using TRIzol reagent (TR118, MRC,USA). Prior to isolation of extracellular RNA (exosomes), 25 fmol of *C. elegans* cel-mir-39 standard RNA (Ribobio, Guangzhou, China) was added to each sample as a spike-in control [25–27]. Before isopropanol precipitation, Dr.GenTLE Precipitation Carrier (TAKARA#9094, RR820A, Takara, Japan) was added as a co-precipitant to enhance the yield of extracellular RNA.

### Exosome treatment

Exosomes were isolated from 5 × 10^6^ normoxic or hypoxic mBMSCs and hBMSCs, Cells were planted into 6-well plates one day before treatment. When the cells grew at about 70% of confluent, 200μg of exosomes were directly added into cells. PBS was added as control. Forty-eight hrs after treatment, cells were collected for the following experiments.

### Blockade of exosome generation by GW4869

GW4869 (Sigma, St. Louis, MO, USA) was used as an inhibitor of exosomes biogenesis/release. GW4869 was added into the medium with 10% exosome-free FBS before BMSCs were put in hypoxic chamber. 3 days after hypoxic treatment,the conditioned medium of MSCs were collected for exosome isolation as mentioned above.

### MiRNA microarray of exosomes

Plasma exosomes from mice that received co-injection of BMSCs and LLC cells or injection of LLC cell alone and exosomes from hypoxia-treated mBMSCs or normoxia-treated mBMSCs were collected for microarray analysis. Agilent Mouse miRNA microarray (v19.0; Agilent Technologies Inc., Santa Clara, CA, USA) was used in the analysis. MiRNAs were labeled and hybridized with miRNA Complete Labeling and Hybridization kit (Agilent Technologies) according to the manufacturer’s protocol. The original data files were processed by Feature Extraction software. Signals were normalized using Gene Spring GX software 11.0 (Agilent Technologies).ANOVA was used to compare the different miRNA expressions. The microarray data have been submitted to the Gene Expression Omnibus and the data could be accessed by the accession numbers GSE119887 and GSE119790.

### RNA sequencing

C57BL/6 mice were subcutaneously injected with LLC-RFP with or without BMSCs. When the size of tumours reached 150–200 mm^3^, the red fluorescent protein positive LLC cells were collected from the tumour sites by flow cytometry cell sorting and subjected to RNA sequencing analysis. The total RNA was isolated from the cell using TRIzol reagent (Life Technologies, Carlsbad, CA) according to the manufacturer’s instructions. The extracted RNA was then quantified and assessed for integrity using the NanoDrop (Thermo, USA). The sample quality control, library preparation and sequencing were performed by BGI, China. Briefly, library preparation was performed using oligo-dT beads for enrichment with mRNA containing poly-A tails. RNA was then fragmented and reversely transcribed to double-stranded cDNA (dscDNA) using random hexamer primers. These cDNA fragments then have the addition of a single ‘A’ base and subsequent ligation of the adapter. Then quantified the PCR products by Qubit and pooled samples together to make a single strand DNA circle (ssDNA circle), which gave the final library. Each library was then sequenced at a depth of 10G clean reads on the BGISEQ500 platform (BGI, China). The dscDNA was then end-repaired and ligated to the bubble adapter with protruding T of 3′ end. During PCR amplification step, the fragments were separated into single strands, amplified, and cyclized to form ‘DNA nanoballs’. The DNA nanoballs were loaded into the patterned nanoarrays and pair-end reads of 100 bp were read through on the BGISEQ-500 platform for the following data analysis study. The RNA sequencing data have been submitted to the Gene Expression Omnibus and the data could be accessed by the accession numbers GSE120349.

### Cellular internalization of exosomes

Exosomes isolated from culture medium were labelled with the green-fluorescing, lipophilic dye PKH67 according to the manufacturer’s recommendations (Sigma, St. Louis, MO, USA) [28]. Briefly, exosomes were resuspended in 1 mL Diluent C mixed with 4 μL PKH67 and then incubated for 4 mins at room temperature. An equal volume of exosome-free FBS was added to stop the reaction before repelleting. Exosomes were washed twice with exosome-free FBS/ RPMI-1640 to remove excess PKH67. Labelled exosomes were resuspended in 1 × PBS and used immediately or stored at − 20 °C.

Fluorescently labelled exosomes prepared above were resuspended and added to LLC-RFP cells that were at 80% confluence, and incubated for 5 h. Cells were washed twice with PBS, fixed with 4% paraformaldehyde for 30 min at room temperature. The cells were then washed with PBS for three times and stained with mounting medium containing 4′,6-diamidino-2-phenylindole (DAPI, Vector, Shield, Burlingame, CA) for 5 mins. After staining, cells were washed twice with PBS to remove excess DAPI. LLC-RFP cells that uptook the labelled exosomes were observed under a fluorescence microscope (BX53, Olympus, Japan).

### Cell migration and invasion assays

Transwell migration assay was performed using a transwell insert that contains polycarbonate filters with 8-μm pores (cat. no. 3422; Corning).Transfected cells (5 × 10^4^) were suspended in 200 μl of serum-free medium and added to the transwell membrane in the upper chamber. Exosomes that were suspended in complete medium were added to the lower chamber. Cells were incubated at 37 °C for 24 h to allow cell migration through the membrane. Migrated cells were fixed in 4% paraformaldehyde and stained with crystal violet. Migrated cell images were observed and imaged under microscope (CKX41, Olympus, Japan). Cell migration was quantitated by counting in 10 random fields on the lower membrane surface.

Invasion capacity of cells was measured by Matrigel matrix gel invasion assay using a Transwell system (BD Biosciences, CA, USA). The surface of the filter (8-μm pore size) of the upper chamber was coated with 1 mg/ml Matrigel matrix. Exosomes suspended in complete medium were added to the bottom wells. Twenty four hours after incubation, the non-invasive cells were removed with a cotton swab. Migrated cells were fixed in 4% paraformaldehyde and stained with crystal violet. Cell invasion was observed and imaged under microscope (CKX41, Olympus, Japan). Cell invasion was quantitated by counting in 10 random fields on the lower membrane surface.

### Western blot analysis

The protein lysate used for western blotting was extracted using mRIPA buffer (Biotime, Hangzhou, China) containing protease inhibitors (Roche, Basel, Switzerland). Proteins were quantified using the BCA™ Protein Assay Kit (Pierce, USA).A western blot system was set up using a Bio-Rad Bis-Tris Gel system, according to the manufacturer’s instructions (Bio-Rad, CA, USA). The cell protein lysates were separated on 10% SDS-polyacrylamide gels and electrophoretically transferred to polyvinylidenedifluoride membranes (Millipore, Danvers, MA, USA). The primary antibody solution was prepared in 5% blocking buffer. Primary antibodies against STAT3, p-STAT3 (Cell Signaling Technology, USA) were incubated with the membrane at 4 °C overnight, followed by a brief wash and incubation with secondary antibody for 1 h at room temperature. An anti-β-actin antibody control was purchased from Proteintech (Chicago,USA) and was used as a loading control. Finally, a 40:1 solution of peroxide and luminol was added to cover the blot surface for five minutes at room temperature. The chemiluminescent signals were captured, and the intensity of the bands was quantified using a Bio-Rad ChemiDoc XRS system (Bio-Rad, CA, USA).

### Quantitative real-time PCR

Total RNA was isolated from cells or exosomes using Trizol reagent (Invitrogen, CA, USA).cDNAwas synthesized from total RNA using the RevertAid First Strand cDNA Synthesis Kit (Thermo Scientific, Waltham, MA, USA) and miDETECT A TrackTM miRNA qRT-PCR starter Kit (Ribobio, Guangzhou, China). U6 snRNA was used as an endogenous control to normalize miRNA expression in cells, and cel-miR-39 was used to normalize miRNA expression in exosomes between the samples. β-actin was used as the endogenous control to normalize expressions of EMT related molecules. The primers of miRNAs were synthesized by RiboBio Company, and the primers of EMT related molecules including E-cadherin, N-cadherin, Vimentin, Fibronectin, Snail, Twist, Zeb1 and Slug were synthesized by Sangon Biotech (Shanghai, China). The sequences were presented in Table 2. Quantitation PCR was performed according to the indications. Real-time PCR was performed using the Bio-Rad IQ™^5^ Multicolor Real-Time PCR detection System (Bio-Rad, Berkeley, CA, USA).

**Table 2:** RT-PCR primers

Gene	Forward primer (5′- to 3′)	Reverse primer (5′- to 3′)
E-cadherin	CAGGTCTCCTCATGGCTTTGC	CTTCCGAAAAGAAGGCTGTCC
N-cadherin	AGCGCAGTCTTACCGAAGG	TCGCTGCTTTCATACTGAACTTT
Vimentin	CGTCCACACGCACCTACAG	GGGGGATGAGGAATAGAGGCT
Fibronectin	ATGTGGACCCCTCCTGATAGT	GCCCAGTGATTTCAGCAAAGG
Snail	CACACGCTGCCTTGTGTCT	GGTCAGCAAAAGCACGGTT
Twist	GGACAAGCTGAGCAAGATTCA	CGGAGAAGGCGTAGCTGAG
Zeb1	GCTGGCAAGACAACGTGAAAG	GCCTCAGGATAAATGACGGC
Slug	TGGTCAAGAAACATTTCAACGCC	GGTGAGGATCTCTGGTTTTGGTA
β-actin	GTGCTATGTTGCTCTAGACTTCG	ATGCCACAGGATTCCATACC

The primers of RNU6 and cel-miR-39 were purchased from Guangzhou RiboBio Co.,Ltd. (Guangzhou, China). Relative miRNA and mRNA expression levels were calculated by the 2-ΔΔCt method. All samples were tested thrice.

### Cell transfection

miR-193a-3p and miR-210-3p or miR-5100 inhibitors and mimics (2′-O-methyl modification) were synthesized by RiboBio Company. Lung cancer cells were seeded into six-well plates 24 h before transfection. When cells reached 50% confluency, miRNA mimics (50 nM) or miRNA inhibitor (100 nM) was transfected with Lipofectamine 3000 according to the manufacturer’s instructions(Life Technologies). Briefly, 3 μl of lipofectamine 3000 reagent was diluted in 100 μl of opti-MEM medium. 5 μl of mimics/10 μl of inhibitors were diluted in 100 μl of opti-MEM medium. Upon mixing above diluted reagents, the mixture was incubated at room temperature for 20 mins and added to the cells. Exosomes were added immediately after inhibitor transfection mixture addition. 48 h after transfection, cells were harvested for the following experiments.

### Animal experiments

Six-week-old female C57BL/6 mice were used to examine allograft tumor growth. All animal experiments were conducted following protocols approved by Central South University, China. A total of 1 × 10^6^ of murine Lewis Lung Cancer LLC-luciferase cells with or without murine BMSCs were injected subcutaneously into mice. Animals were randomly divided into four groups. When the mean tumor volume reached 100mm^3^, hypoxic mBMSC-secreted exosomes or normoxic mBMSC-secreted exosomes were intratumorally injected into mice at a dose of 200μg per mouse every 2 days for 10 times. Lung metastasis were measured by H&E staining on lung paraffin sections, or detected by ex vivo luciferase based noninvasive bioluminescence imaging using IVIS Lumina II (PerkinElmer). Artagain black paper (Strathmore, USA) was used to cover the primary tumours to avoid signal saturation.

### Statistical analysis

All statistical analyses were performed using the SPSS 18.0 software, and the graphs were generated using GraphPad Prism 6.0 (GraphPad Software, San Diego, CA, USA). The comparisons of means among groups were analyzed by one way ANOVA, and the Dunn Multiple Comparison Test was further used to determine significant difference between groups. Receiver operating characteristic (ROC) curves and the area under the ROC curve (AUC) were established to determine the sensitivity and specificity of the miRNAs for the prediction of tumorigenesis and metastasis in the patients. Logistic regression was used to develop a combined miRNA panel to predict the probability of developing tumours as previously described [29]. Spearman’s correlation analysis was conducted to evaluate whether the expression levels of the 2 miRNAs were correlated with one another and with other common clinical biomarkers. A value of *P* < 0.05 was considered statistically significant.

## Results

### Identifying exosomal miRNAs secreted from hypoxic BMSCs that improve the invasion of lung cancer cells

**Fig. 1:**
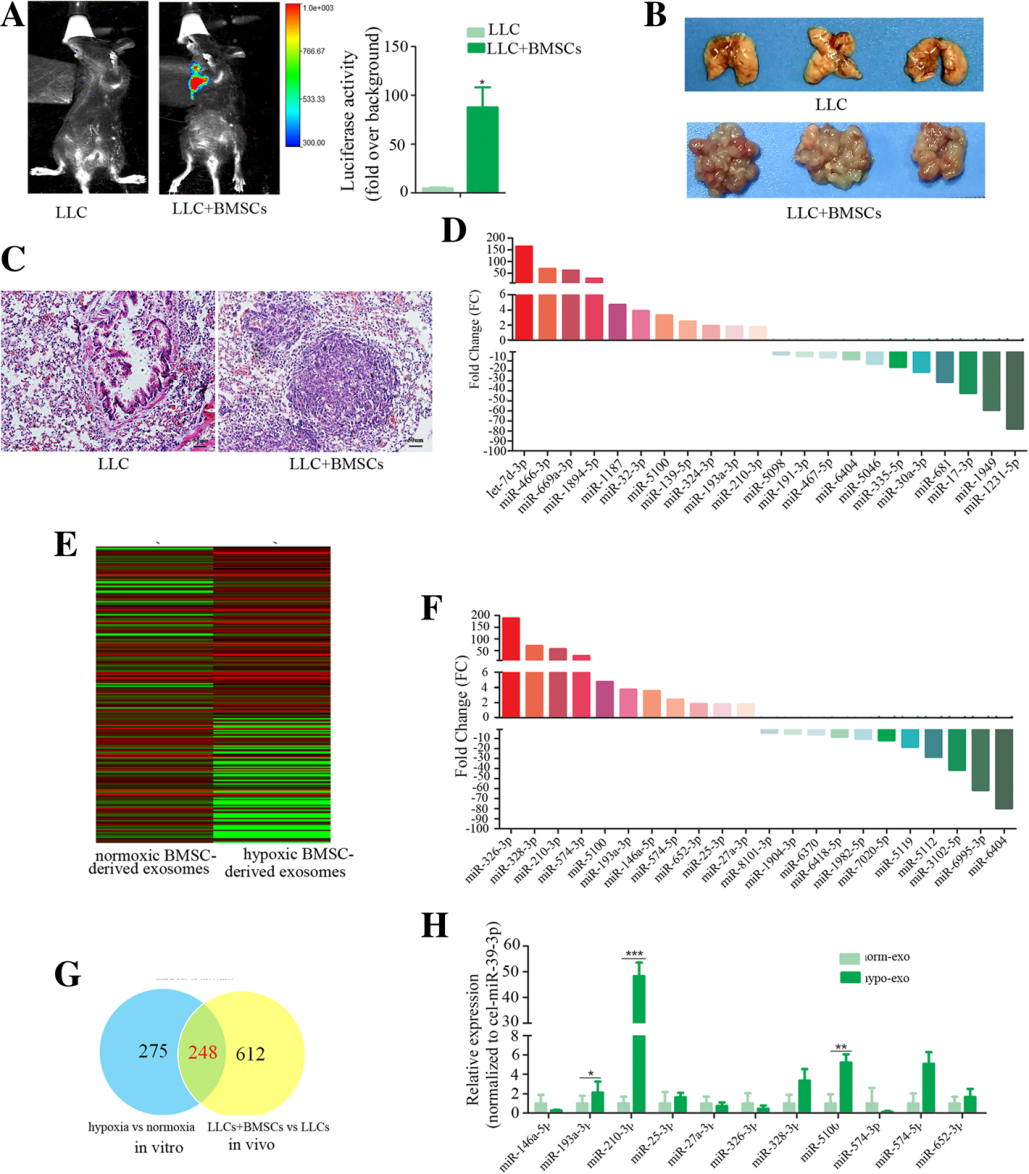
Identifying exosomal miRNAs secreted from hypoxic BMSCs that improve the invasion of lung cancer Cells. LLC cells that were stably transfected with the firefly luciferase gene (luci-LLC) were subcutaneously injected with or without BMSCs into C57BL/6 mice. 28 days after treatment, the bioluminescence imaging system was used to monitor the mobility and growth of LLC cells in vivo. **a** Bioluminescent images were shown. The activities of luciferase were shown in histogram. **b** Representative images of lung metastatic nodules from mice. **c** Representative images of Hematoxylin and Eosin (HE) staining of lungs section. HE staining was performed to detect the metastatic nodules in the lung. **d** Representative upregulated or downregulated plasma exosomal microRNAs from mice that received co-injection of BMSCs and LLC cells in contrast to mice that received injection of LLC cells alone. **e** The BMSCs were cultured in a hypoxic chamber for 3 days. The exosomes were collected and isolated from the cell culture medium. The exosomal miRNA expression profile was examined.Hierarchical clustering analysis of exosomal microRNA expression. Signals were normalized using Gene Spring GX software 11.0. Clustering was performed on differentially expressed exosomal microRNAs between hypoxic BMSC-secreted exosomes and normoxic BMSC-secreted exosome. Columns represent individual samples and rows represent each exosomal microRNA. Red and green in cells reflect high and low expression levels, respectively, as indicated in the scale bar (log2-transformed scale). **f** Representative upregulated or downregulated microRNAs from hypoxic BMSC- secreted exosomes compared to normoxic BMSC-secreted exosomes. **g** Venn diagram showed the overlap of shared exosomal miRNAs in vitro and in vivo. **h** Expression of selected exosomal miRNAs was verified by quantitative real-time PCR. Experiments were performed in triplicate. Student’s t-test, *p < 0.05, **p < 0.01, ***p < 0.001

It has long been known that hypoxia is the main feature of cancer microenvironments. BMSCs in cancer microenvironments contribute to cancer progression. We first determined the effects of BMSCs on tumor cell metastasis in a syngeneic Lewis lung carcinoma (LLC) mouse model. LLC cells that were stably transfected with the firefly luciferase gene (luci-LLC) were subcutaneously injected alone or with BMSCs into C57BL/6 mice. The mice were administrated with same amount of LLC cells. The mice that received co-injection of LLC cells and BMSCs developed larger allograft tumours compared to the LLC cell injection alone (Additional file 1: Figure S1A). The mobility and growth of the LLC cells in vivo were examined by bioluminescence imaging. In contrast to the mice that received injection of LLC cells alone, which had little evidence of micrometastatic cells, the mice that received injection of a mixture of LLC cells and BMSCs showed high luciferase activity in the lungs (Fig. 1a). The number of metastatic tumor nodules formed in the lungs was greater in the mixture-injection group than in the LLC single-injection group (Fig. 1b, Additional file 1: Figure S1B). Hematoxylin and eosin-stained sections showed more metastatic nodules in the lungs in the mixture-injection group (Fig. 1c). These results indicate that co-injection of BMSCs and LLCs into syngeneic mice leads to increased growth of the primary cancer and metastasis to lung. We next examined the plasma exosomal miRNA profile of mice that received injection of a mixture of BMSCs and LLC cells or LLC cells alone. miRNA expression profiling of plasma exosomes revealed that 86 exosomal miRNAs were significantly increased and 354 exosomal miRNAs were significantly decreased in the co-injection group compared to LLC injection alone (Fig. 1d, Additional file 1: Figure S1C).

In addition to BMSCs, other cell types such as cancer cells were also affected by hypoxic microenvironment and released exosomes. We then investigate to what extent hypoxia affects the exosome release of BMSCs. The BMSCs were cultured in a hypoxic chamber for 3 days. The exosomes were collected and isolated from the cell culture medium. The exosomes collected from hypoxic BMSC medium were smaller than those from normoxic BMSC medium, with average diameters of 50 nm and 90 nm, respectively (Additional file 1: Figure S1D). The exosomes were observed by transmission electron microscopy (Additional file 1: Figure S1E). The exosomes were positive for exosomal proteins CD63 and HSP70 (Additional file 1: Figure S1F). We then examined BMSC exosomal miRNA expression profile. Total RNAs were extracted and subjected to miRNA microarray. Some exosomal miRNAs including miR-146a-5p, miR-574-3p, miR-328-3p, miR-326-3p, miR-193a-3p, miR-5100 and miR-210-3p were increased, while others such as miR-6404, miR-6995-3p, miR-5112 were decreased by more than 2-fold in hypoxic BMSCs compared to normoxic BMSCs (Fig. 1e and f). We identified overlap of shared exosomal miRNAs in vitro and in vivo that are specific exosomal miRNAs derived from BMSCs (Fig. 1g). The expression of microRNAs was verified by qPCR, showing that exo-miR-210-3p, exo-miR-328-3p, exo-miR-574-5p, exo-miR-25-3p, exo-miR-652-3p, exo-miR-193a-3p and exo-miR-5100 secreted by hypoxic BMSCs were upregulated compared to normoxic BMSCs secreted exosomes (Fig. 1h). We selected exo-miR-193a-3p, exo-miR-210-3p and exo-miR-5100, which showed similar expression pattern in vitro and in vivo for further investigation.

### Exosome-mediated transfer of miR-193a, miR-210-3p and miR-5100 from hypoxic BMSCs promotes invasion of cancer cells

**Fig. 2:**
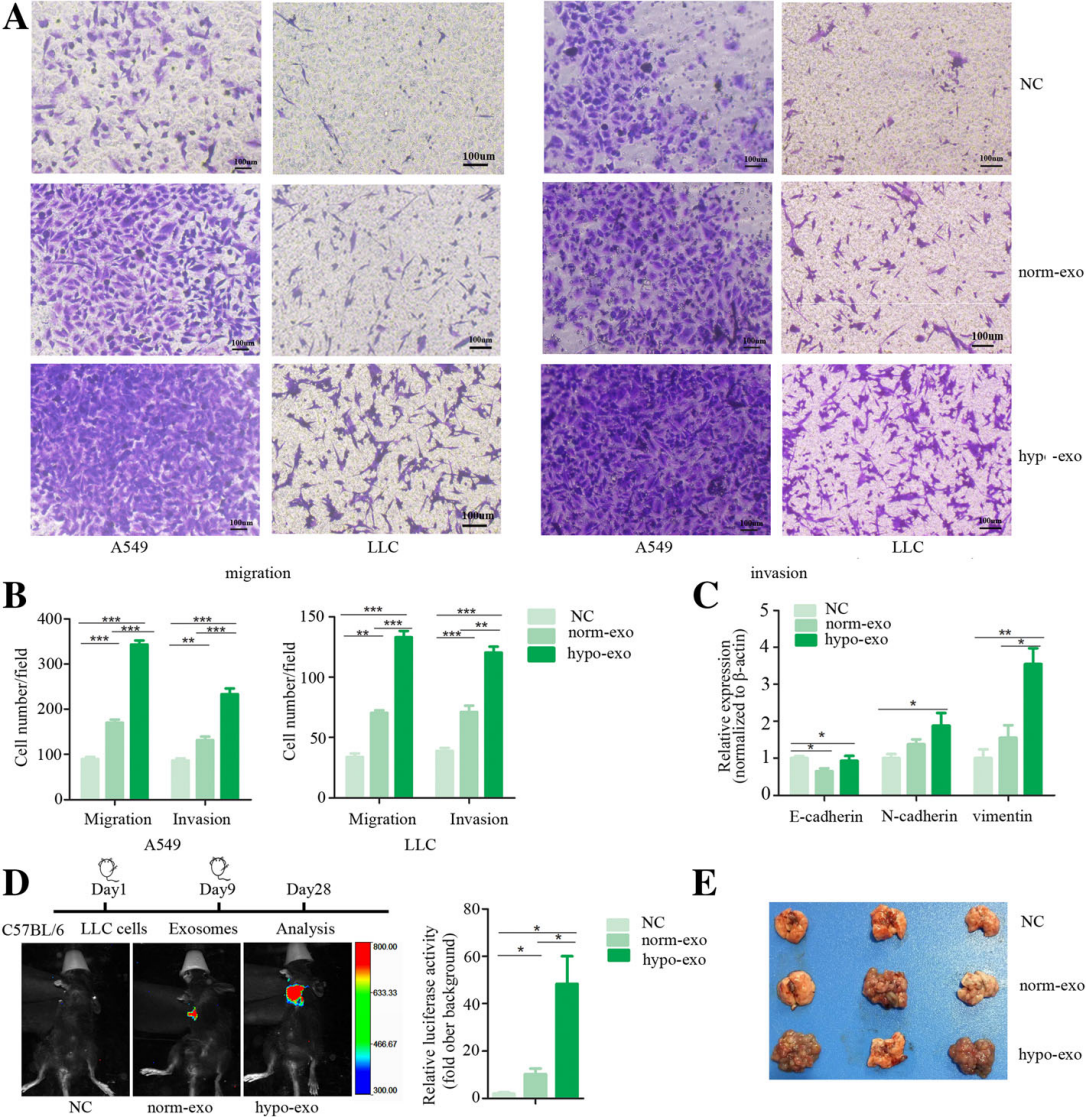
Hypoxic-BMSC-derived exosomes promote metastasis of lung cancer cells via EMT. **a** Cell migration and invasion were measured by transwell assays. A549 cells and LLC cells were treated with hypoxic BMSC-secreted or normoxic BMSC-secreted exosomes for 48 h. Cells that migrated or invaded to the bottom surface were stained with crystal violet and observed by light microscopy (magnification, 100×). **b** The numbers of migrating cells or invading cells were counted from six fields of view in each group. Data were presented as the mean ± SD, and analyzed with Student’s t-test. *P < 0.05; ** < 0.01; *** < 0.001. **c** Epithelial and mesenchymal markers in exosome-treated lung cancer cells were measured by quantitative real-time PCR. Experiments were performed in triplicate. *P < 0.05; ** < 0.01. **d** Bioluminescent images were shown. Timeline of experimental protocol was shown above. C57BL/6 mice were subcutaneously injected with LLC-luciferase cells. When the mean tumor volume reached 100 mm^3^, the mice were intratumorally injected with exosomes released from hypoxic BMSCs or normoxic BMSCs. Activities of luciferase were shown in histogram. **e** Representative images of metastatic tumor nodules formed in the lungs

We next studied the roles of hypoxic BMSC-derived exosomes in the invasion of lung cancer cells. A549, H358, H460 and LLC cells were treated with exosomes and cellular invasion was evaluated by a migration and invasion transwell assay. It revealed that hypoxic BMSC-secreted exosomes promote the migration and invasion of lung cancer cells compared to normoxic BMSC-secreted exosomes (Fig. 2a and b, Additional file 2: Figure S2A and B). The cancer cells treated with hypoxic BMSC-derived exosomes displayed epithelial mesenchymal transition (EMT), transforming into spindle-shaped mesenchymal-like cells(Additional file 2: Figure S2C). We examined epithelial and mesenchymal markers in exosome-treated lung cancer cells. The mesenchymal markers vimentin and N-cadherin were higher and the epithelial marker E-cadherin was lower after treatment with hypoxic BMSC-secreted exosomes compared to normoxic BMSC-secreted exosomes (Fig. 2c). The effects of hypoxic BMSC-derived exosomes on tumor cell metastasis were evaluated in vivo. C57BL/6 mice were subcutaneously injected with LLC-luciferase cells. When the mean tumor volume reached 100 mm^3^, the mice were intratumorally injected with exosomes released from hypoxic BMSCs or normoxic BMSCs. Hypoxic BMSC-secreted exosomes greatly improved the invasion of cancer cells and improved metastasis of cancer cells to the lung(Fig. 2d). The number of metastatic tumor modules formed in the lungs was greater in the mice that received hypoxic BMSC-derived exosomes than in the mice that received normoxic BMSC-derived exosomes or in the control group (Fig. 2e, Additional file 2: Figure S2D).

**Fig. 3:**
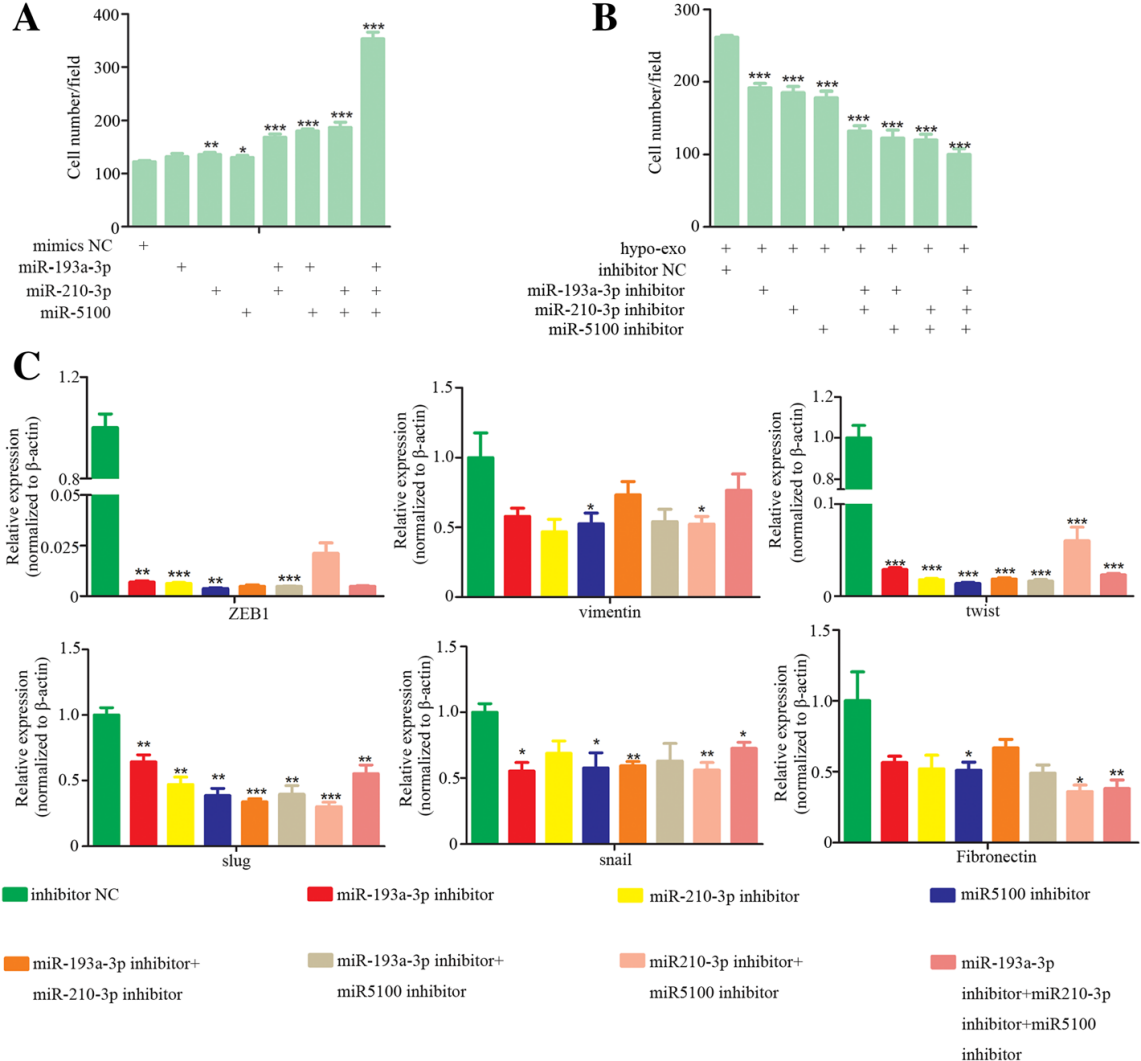
Exosome-mediated transfer of miR-193a, miR-210-3p and miR-5100 from BMSCs promotes invasion of cancer cells. **a** Cell invasion were measured by transwell assays. Cells were transfected with mimics of miR-193a-3p, miR-210-3p and miR-5100. Cells that invaded to the bottom surface were stained with crystal violet and observed by light microscopy (magnification, 100×). The numbers of invading cells were counted from six fields of view in each group. Data were presented as the mean ± SD, and analyzed with Student’s t-test. *P < 0.05; ** < 0.01; *** < 0.001. **b** Cell invasion were measured by transwell assays. LLC cells were treated with hypoxic BMSC-secreted exosomes followed by transfection with inhibitors of miR-193a-3p, miR-210-3p and miR-5100. Cells that invaded to the bottom surface were stained with crystal violet and observed by light microscopy (magnification, 100×). The numbers of migrating cells or invading cells were counted from six fields of view in each group. Data were presented as the mean ± SD, and analyzed with Student’s t-test. *P < 0.05; ** < 0.01; *** < 0.001. **c** The expression of epithelial and mesenchymal markers in LLC cells measured by quantitative real-time PCR. LLC cells were treated with hypoxic BMSC-secreted exosomes followed by transfection with inhibitors of miR-193a-3p, miR-210-3p and miR-5100. Experiments were performed in triplicate. Data were presented as the mean ± SD, and analyzed with Student’s t-test. *P < 0.05; ** < 0.01; *** < 0.001

We next determined whether the exosome-mediated transfer of select miRNAs from BMSCs contributes to cancer cell invasion. Transduction mimics of miR-193a-3p, miR-210-3p and miR-5100 enhanced the cancer cell invasion, and the combination of these microRNAs enhanced cancer cell invasion further (Fig. 3a, Additional file 3: Figure S3A). A miR-193 inhibitor, miR-210-3p inhibitor and miR-1500 inhibitor were transfected into cancer cells before treatment with hypoxic BMSC-secreted exosomes. We found that these inhibitors could reverse the enhanced invasion induced by exosomes released from hypoxia and inhibit the expression of mesenchymal related molecules such as vimentin, slug, snail, twist, fibronectin and ZEB1 (Fig. 3b and c, Additional file 3: Figure S3B).

**Fig. 4:**
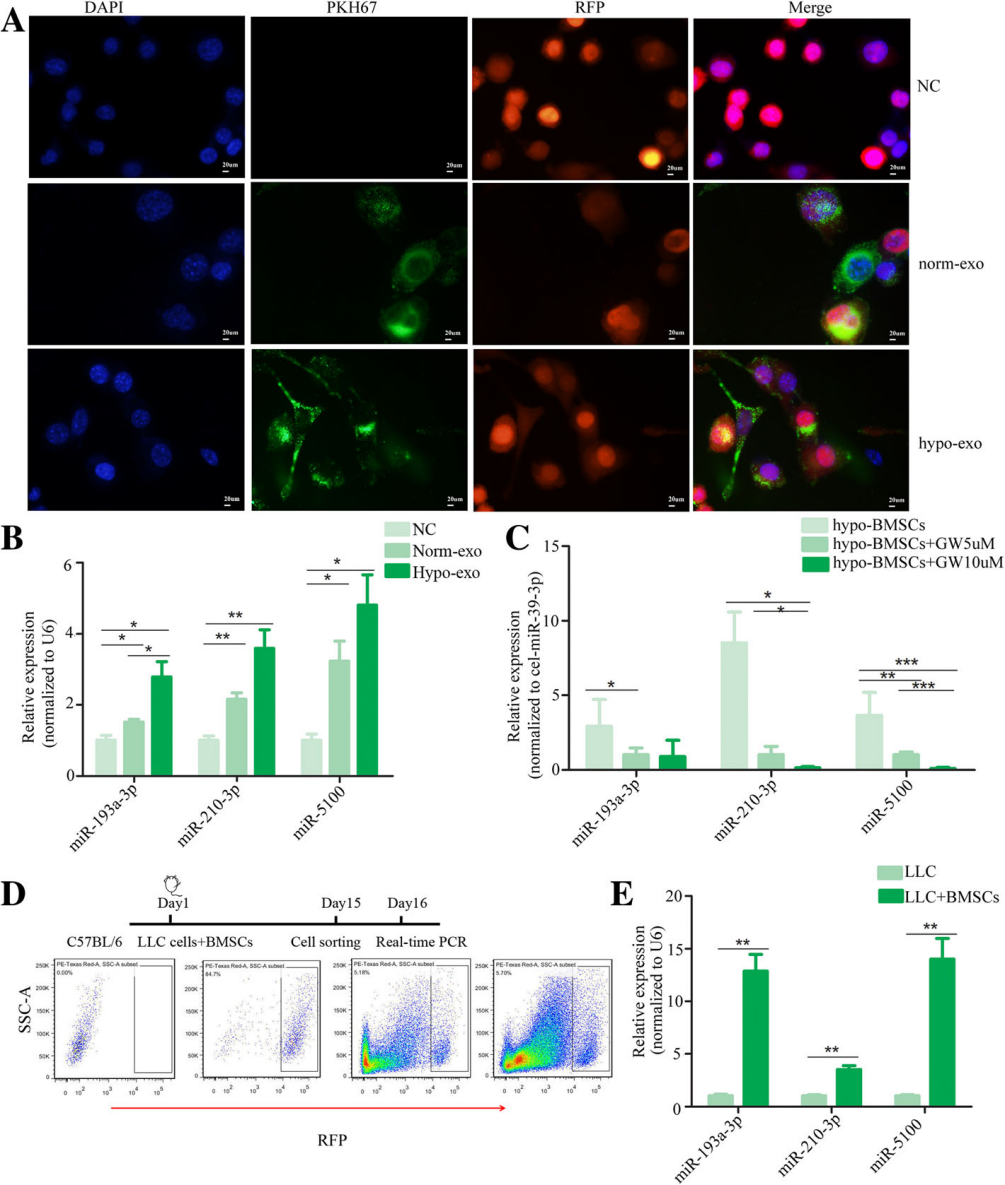
The exosomal miRNAs were transferred from BMSCs to the cancer cells. **a** Fluorescently labeled exosomes entered into LLC cells. Representative images were filmed after cells were fixed and stained (magnification, 400×). **b** The expression of miR-193a-3p, miR-210-3p and miR-5100 in cancer cells after treatment measured by Quantitative real-time PCR. LLC cells were treated with normoxic or hypoxic BMSC-secreted exosomes. Experiments were performed in triplicate.*P < 0.05. **c** The expression of exosomal miR-193a-3p, miR-210-3p and miR-5100 measured by Quantitative real-time PCR. Exosomal microRNAs were isolated from hypoxic BMSCs that treated with GW4869. Experiments were performed in triplicate Student’s t-test. *P < 0.05; ** < 0.01; *** < 0.001. **d** LLC cells were isolated by flow cytometry cell sorting. Timeline of experimental protocol was shown above. C57BL/6 mice were subcutaneously injected with LLC-RFP with or without BMSCs. When the mean tumor volume reached 150–200 mm^3^, the red fluorescent protein positive-LLC cells were collected from the tumor sites by flow cytometry cell sorting. **e** The levels of miR-193a-3p, miR-210-3p and miR-1500 in sorted LLC cells measured by quantitative real-time PCR. Experiments were performed in triplicate. Student’s t-test. *P < 0.05; ** < 0.01; *** < 0.001

We then examined whether exosomes secreted by BMSCs translocated into cancer cells. The exosomes were labeled with PKH67 (Sigma, USA) and were incubated with cancer cells. As shown in Fig. 4a, the fluorescent exosomes entered the cancer cells. miR-193a-3p, miR-210-3p and miR-1500, carried by exosomes, were increased in the cancer cells after treatment hypoxic BMSC-derived exosomes (Fig. 4b). The treatment with hypoxic BMSC medium also resulted in the upregulated expression of miR-193a-3p, miR-210-3p and miR-5100(Additional file 4: Figure S4A). The cellular expression miR-193a-3p, miR-210-3p or miR-5100 of hypoxic BMSCs was less than that of nomoxic BMSCs, indicating that most of these microRNAs are assembled on the exosomes and secreted outside of cells (Additional file 4: Figure S4B). When the BMSCs were treated with GW4869, these microRNAs in exosomes from BMSCs were significantly decreased (Fig. 4c). C57BL/6 mice were subcutaneously injected with LLC-RFP with or without BMSCs. When the mean tumor volume reached 150–200 mm^3^, the red fluorescent protein positive-LLC cells were collected from the tumor sites by flow cytometry cell sorting (Fig. 4d). The levels of miR-193a-3p, miR-210-3p and miR-1500 were significantly higher in these LLC cells collected from the co-injection group, indicating that these miRNAs were transferred from BMSCs to the cancer cells (Fig. 4e).

### Exosomal miRNAs from BMSCs promote metastasis by STAT3 driven EMT

**Fig. 5:**
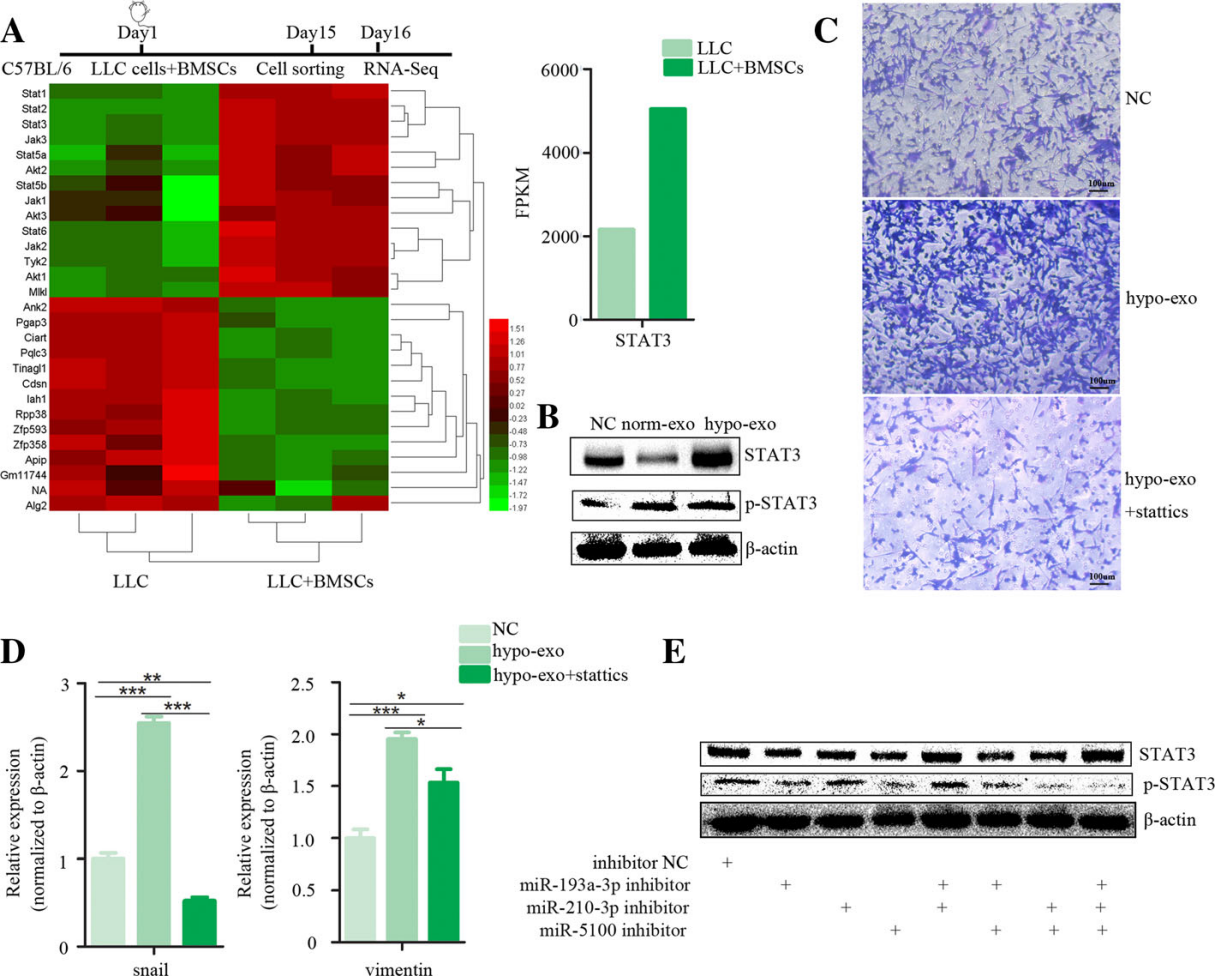
Exosomal miRNAs from BMSCs promote metastasis by STAT3 driven EMT. C57BL/6 mice were subcutaneously injected with LLC-RFP with or without BMSCs. The red fluorescent protein positive LLC cells were collected from the tumor sites by flow cytometry cell sorting and subjected to RNA sequencing analysis. Timeline of experimental protocol was shown. **a** The upregulated genes were enriched in the JAK-STAT pathway. Clustering was performed on differentially expressed mRNAs between LLC cells collected from mice that received co-injection of BMSCs and LLC or LLC injection alone. Columns represent individual samples and rows represent each gene. Red and green reflect high and low expression levels, respectively. FPKM for *STAT3* transcripts obtained by RNA-Seq was shown in histogram. **b** Protein expression of STAT3, p-STAT3 were measured by Western blot analysis. Cells were treated with normoxic BMSC-secreted exosomes or hypoxic BMSC-secreted exosomes. β-actin was used as the internal control. **c** Cell invasion was measured by transwell assay. The increased invasion capability induced by hypoxic BMSC-secreted exosomes was reversed by stat3 inhibitor stattics. Magnification, 100×. **d** Expression of Snail and Vimentin was measured by quantitative real-time PCR. Cells were treated with hypoxic BMSC-secreted exosomes with or without stat3 inhibitor stattics. Experiments were performed in triplicate.*P < 0.05. **e** Expression of stat3 measured by western blot. The miR-193a-3p inhibitor, miR-210-3p inhibitor and miR-5100 inhibitor could reverse the enhanced expression of phosphorylated STAT3 induced by exosomes released from hypoxia

To uncover the mechanism driving the BMSC pro-tumorigenic effect on cancer cells, we analyzed transcriptomic profiles in LLC cells isolated from primary cancer sites. C57BL/6 mice were subcutaneously injected with LLC-RFP with or without BMSCs. When the size of tumours reached 150–200 mm^3^, the red fluorescent protein positive LLC cells were collected from the tumor sites by flow cytometry cell sorting and subjected to RNA sequencing analysis. Gene expression profiling was performed on LLCs collected from 3 samples for each group. The global gene expression profile of LLC cells collected from tumours formed by LLC cells alone is different from cells collected from tumours formed by co-injection with LLC cells and BMSCs (Additional file 5: Figure S5A). Compared to LLC cells collected from tumours in LLC injection alone, LLCs isolated from co-injected mice had 1330 upregulated and 1037 downregulated genes. The upregulated genes were enriched in the JAK-STAT pathway (Fig. 5a). We further found that the exosomes secreted from hypoxic BMSCs increased the cancer cell expression of total and phosphorylated STAT3 (Fig. 5b). The stat3 inhibitor stattics inhibited the cancer cell invasion and the expression of mesenchymal markers snail and vimentin was induced by hypoxic BMSC-secreted exosomes (Fig. 5c and d, Additional file 5: Figure S5B). We then examined whether exosomal miRNAs regulate the JAK-STAT pathway. The miR-193a-3p inhibitor, miR-210-3p inhibitor and miR-5100 inhibitor could reverse the enhanced expression of phosphorylated STAT3 induced by exosomes released from hypoxia, indicating that the activation of STAT3 by exosomes was induced by microRNAs carried by exosomes (Fig. 5e).

### Exosomal miRNA can be biomarkers for lung cancer metastasis

**Fig. 6:**
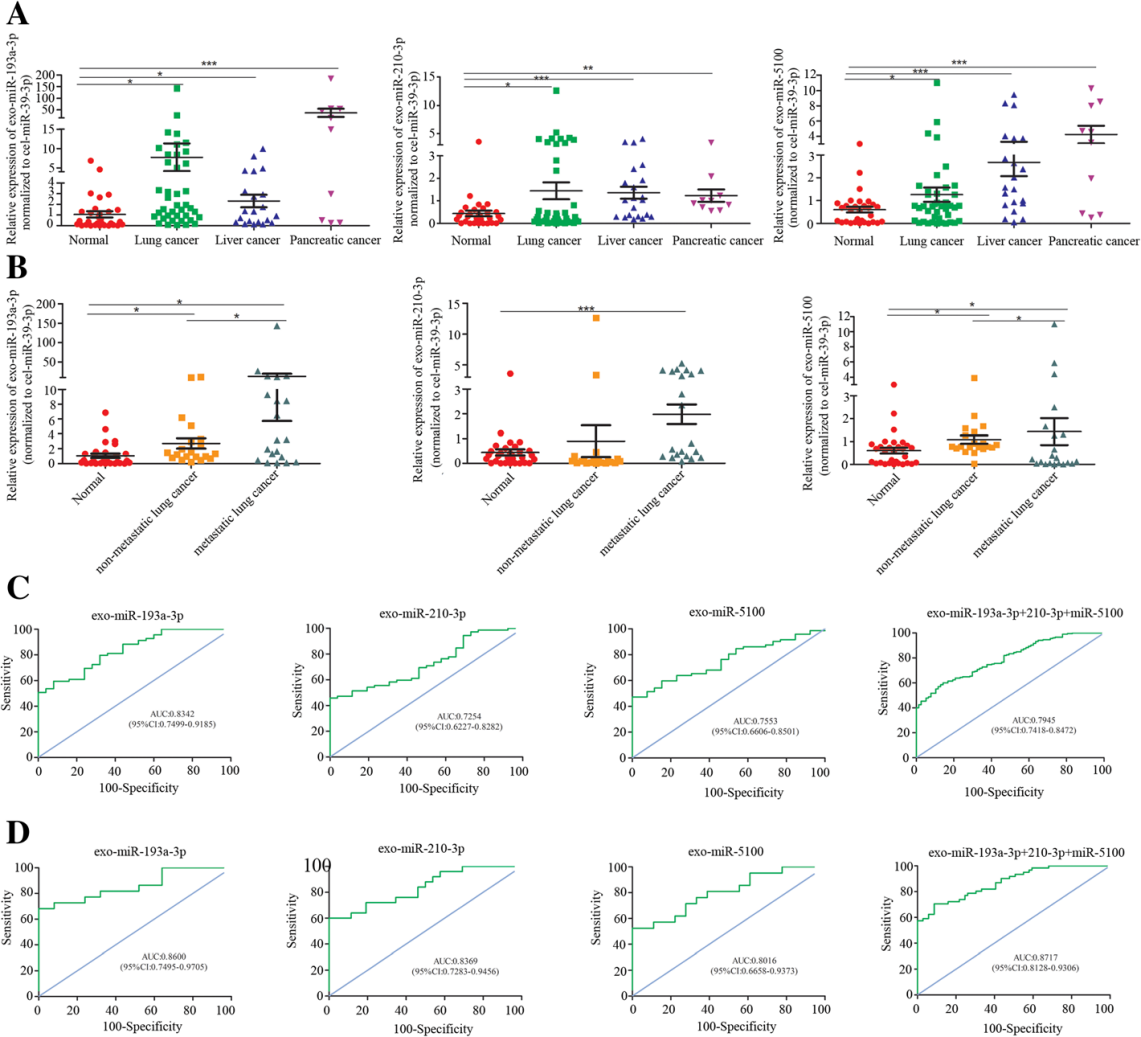
Exosomal miRNA can be biomarkers for cancer metastasis. Plasma samples were collected from 72 patients with pancreatic cancer, lung cancer or liver cancer and 30 healthy controls. Plasma exosomes were isolated. **a** Expression of miR-193a-3p and miR-210-3p and miR-5100 in plasma exosomes from the lung cancer patients (*n* = 41),liver cancer patients(*n* = 21),pancreas cancer patients(*n* = 10),and normal healthy control (*n* = 30). **b** Expression of miR-193a-3p and miR-210-3p and miR-5100 in plasma exosomes from the metastatic lung cancer patients (*n* = 21),non-metastatic lung cancer patients (*n* = 20) and normal samples (*n* = 30). **c** ROC analysis of the individual exosomal miRNA and the three exosomal miRNA panel for cancer patients and non-cancerous control. **d** ROC analysis of the individual exosomal miRNA and the three exosomal miRNAs panel for the metastatic lung cancer patients and non-metastatic lung cancer patients

We further assessed exosomal miR-193-3p, miR-210-3p and miR-5100 by real-time PCR in the cohort consisting of plasma exosomes collected from 72 patients with pancreatic cancer, lung cancer or liver cancer and 30 healthy controls. Clinical characteristics of these cohorts are presented in Table 1. Compared to non-cancerous healthy controls, exosomal miR-193-3p, miR-210-3p and miR-5100 were significantly upregulated in cancer patients (Fig.6a). Plasma exosomal miR-193a-3p, miR-210-3p and miR-5100 in the metastatic lung cancer patients were all significantly upregulated compared to non-metastatic lung cancer patients (Fig. 6b). ROC curve analyses for each exosomal miRNA and the 3 exosomal miRNAs combined were performed. The AUC value for individual miRNAs ranged from 0.7499 to 0.9185 for exosomal miR-193a-3p, 0.6227 to 0.8282 for miR-210-3p, and 0.6606 to 0.8501 for exosomal miR-5100 in discriminating cancer patients from non-cancerous controls (Fig. 6c). ROC analyses yielded AUC values of 0.8600 for miR-193a-3p, 0.8369 for miR-210-3p and 0.8016 for miR-5100 in discriminating metastatic lung cancer patients from non-metastatic lung cancer patients(Fig. 6d). It demonstrated that each individual exosomal miRNAs has the ability to differentiate the cancer patients from non-cancerous control or metastatic lung cancer patients from non-metastatic lung cancer patients (Fig. 6c and d). We found that these 3 exosomal miRNAs showed a higher specificity and sensitivity in discriminating metastatic lung cancer patients from non-metastatic lung cancer patients than in discriminating cancer patients from non-cancerous controls(Fig. 6c and d). Any combination of 2 exosomal miRNAs did not show higher specificity and sensitivity in discriminating cancer patients from non-cancerous control or metastatic lung cancer patients from non-metastatic lung cancer patients (Additional file 6: Figure S6). The combined 3 exosomal miRNAs produced an AUC of 0.8717 in discriminating metastatic lung cancer patients from non-metastatic lung cancer patients; showing a higher specificity and sensitivity (Fig. 6d).

## Discussion

Exosomes are the major mediators of cell–cell communication because they can be captured by neighboring cells [7]. The direct transfer of exosomal miR-193a-3p, miR210-3p and miR-5100 from hypoxic BMSCs to neighboring cancer cells enhances metastatic properties.

BMSCs are multipotent stromal cells that are recruited to tumours and contribute to cancer progression. The effect of hypoxia on BMSCs has been extensively investigated. In this study, we focused on the paracrine effect exosomes secreted by hypoxic BMSCs on the cancer cells. Exosomes are released by most cell types. In order to identify hypoxic BMSC-derived exosomes, we selected overlap of shared exosomal miRNAs in vitro and in vivo. The composition of exosomal microRNAs differs significantly depending on different contexts, indicating selectivity for their loading into exosomes [30]. The proportion of miRNA is higher in exosomes than in their donor cells [2, 31]. RNA binding proteins (RBPs) serve as key players on biosynthesis of exosomes by forming complexes with RNAs and transporting them into exosomes. The heterogeneous nuclear ribonucleoproteins (*hnRNPs*) are a complex and diverse family of RNA binding proteins. hnRNP-dependent pathways are reported to be related to miRNA secretion into exosomes. NSMase2 is an enzyme for ceramide generation that is involved in exosome secretion. The higher expression of NSMase2, hnRNPA1 and hnRNPH1 in hypoxia may contribute to the higher proportion of miRNA on the exosomes [32–34]. Potential hypoxia inducible factor (HIF) binding sites were identified in the promoter of hnRNPA1 [35]. On the other hand, the exosomes secreted from hypoxic cells have a larger surface area because of their smaller size, which may carry more proteins or microRNAs. In this study, we found that the 3’ end of miRNA comprises the main exosomal microRNAs, including miR-193a-3p and miR210-3p, verifying that 3’ portion or the 3’ end of the miRNA sequence contains a critical sorting signal. Exosomal miRNA signatures are composed of a distinct set of miRNAs [12, 36, 37]. In this study, after quantile normalization of the data, miRNAs were differentially expressed in hypoxia-treated versus normoxia-treated BMSCs. Exosomes from B cells, dendritic cells, T cells and colorectal cancer cells have been demonstrated to carry miR-193-3p. miR-210-3p is considered to be hypoxia-related and is carried by exosomes secreted from colorectal cancer cells. Exosome-mediated transfer of particular microRNAs from stromal cells to epithelial cancer cells contributes to cancer progression.

The roles of miR-193a-3p, miR-210-3p and miR-5100 in cancer progression have been reported. miR-193a-3p was demonstrated to suppress the proliferation and migration of lung cancer cells and colorectal adenocarcinoma cells by targeting K-ras [38, 39]. miRNA-193a-3p inhibits tumor proliferation migration and chemoresistance in human gastric cancer by regulating the PTEN gene [40]. On the other hand, the level of miR-193a-3p was significantly increased in renal cell carcinoma (RCC) tissues and cell lines. miR-193a-3p increases cell proliferation and migration of RCC cells by targeting the ST3GalIV via PI3K/Akt pathway [41]. Lower expression of miR-193-3p is associated with poor prognosis in colorectal cancer, but better prognosis in oropharyngeal squamous cell carcinoma (OPSCC) [42, 43]. Similar to the controversial roles of miR-193a-3p, miR-210-3p was also suggested to have oncogenic or tumor suppression effects on the development of cancer, depending on the target [44–46]. A higher level of serum miR-210-3p has been detected in people who live in a high-altitude hypoxic environment [47]. A high level of miR-210-3p in urine was also detected in RCC patients [48]. In this study, we found that cellular expression of specific miRNAs were decreased when these miRNAs were enriched in exosomes, suggesting the differential effects of miRNAs inside and outside of the cells.

Emerging evidence has demonstrated the feasibility of circulating miRNAs as robust, non-invasive biomarkers for the diagnosis of colorectal cancer. Due to their presence in the bodily fluids, exosomes are considered candidate biomarkers. The use of a combination of miRNAs as biomarkers, instead of one unique miRNA, has been considered to be a more powerful tool due to the possible overlap in miRNA targeting [49]. A miRNA may dampen or amplify a signal by participating in a negative or positive feedback loop [50]. In this study, we found a combination of miR-193a-3p, miR-210-3p and miR-5100 promotes the activation of the STAT3 pathway. STAT3 signaling is active when miRNAs target the STAT3 inhibitors SOCS or PIAS3 [51]. MiR-210-3p targets the negative regulator of STAT3 signaling, SOCS1 [46]. Thus, we sought to apply a combination of miRNAs as a biomarker to discriminate between cancer and non-cancerous patients, or between metastatic and non-metastatic patients. A combination of miR-193a-3p, miR-210-3p and miR-5100 can discriminate between the groups with high sensitivity and specificity.

In summary, hypoxic BMSC-derived exosomal miR-193a-3p, miR-210-3p and miR-5100 were identified by microRNA array. Exosome-mediated transfer of particular microRNAs, including miR-193a-3p, miR-210-3p and miR-5100, could promote invasion of cancer cells by activating STAT3 signaling-induced EMT. These exosomal miRNAs may be promising noninvasive biomarkers for cancer progression.

## Conclusions

Exosome-mediated transfer of miR-193a-3p, miR-210-3p and miR-5100, could promote invasion of lung cancer cells by activating STAT3 signaling-induced EMT. These exosomal miRNAs may be promising noninvasive biomarkers for cancer progression.

## Additional file

Additional file 1: **Figure S1.** Identifying exosomal miRNAs secreted from hypoxic BMSCs. LLC cells that were stably transfected with the firefly luciferase gene (luci-LLC) were subcutaneously injected with or without BMSCs into C57BL/6 mice. (A) The mice that received co-injection developed larger allograft tumours compared to the LLC cell injection alone. (B) Number of metastatic tumor nodules formed in the lungs was shown in histogram. (C) Hierarchical clustering analysis of plasma exosomal microRNA expression. Signals were normalized using Gene Spring GX software 11.0. Clustering was performed on differentially expressed exosomal microRNAs at FDR < 0.05. Columns represent individual samples and rows represent each exosomal microRNA. Red and green in cells reflect high and low expression levels, respectively, as indicated in the scale bar (log2-transformed scale). The BMSCs were cultured in a hypoxic chamber for 3 days. The exosomes were collected and isolated from the cell culture medium. (D) Size distribution analysis of purified exosomes by DLS (Zetasizer Nano ZS90 instrument, Malvern). (E) transmission electron microscopy images for exosomes(scale bar = 500 nm). (F) Exosomal markers (CD63,HSP70) were analyzed in exosomes and cell lysate by western blotting. β-actin was used as an internal reference. (TIF 1256 kb)

Additional file 2: **Figure S2.** Hypoxic BMSC-derived exosomes promote lung cancer cells migration and invasion. Cell migration and invasion were measured by transwell assays. (A) H358 and H460 Cells were treated with hypoxic BMSC-secreted or normoxic BMSC-secreted exosomes for 48 h. Cells that invaded to the bottom surface were stained with crystal violet and observed by light microscopy (magnification, 100×). (B) The numbers of migrating cells or invading cells were counted from six fields of view in each group. Data were presented as the mean ± SD, and analyzed with Student’s t-test. *P < 0.05; ** < 0.01; *** < 0.001. (C) Representative images of H358 after treatment with exosomes (magnification,100×). (D) Representative images of Hematoxylin and Eosin (HE) staining of lungs section to detect the metastatic nodules in the lung. (TIF 8240 kb)

Additional file 3: **Figure S3.** Exosome-mediated transfer of miR-193a, miR-210-3p and miR-5100 from BMSCs promotes invasion of cancer cells. (A) Cell invasion were measured by transwell assays. Cells were transfected with mimics of miR-193a-3p, miR-210-3p and miR-5100. Cells that invaded to the bottom surface were stained with crystal violet and observed by light microscopy (magnification, 100×). (B) Cell invasion were measured by transwell assays. The invasion phenotype was reversed by select microRNA inhibitors. LLC cells were treated with hypoxic BMSC-secreted exosomes followed by transfection with inhibitors of miR-193a-3p, miR-210-3p and miR-5100. Cells that invaded to the bottom surface were stained with crystal violet and observed by light microscopy (magnification, 100×). (TIF 5905 kb)

Additional file 4: **Figure S4.** Exosome-mediated transfer of miR-193a, miR-210-3p and miR-5100 from BMSCs to cancer cells. (A)The expression of miR-193a-3p, miR-210-3p and miR-5100 in cancer cells after treatment measured by Quantitative real-time PCR. LLC cells were treated with normoxic or hypoxic BMSC medium. Experiments were performed in triplicate. *P < 0.05; ** < 0.01; *** < 0.001. (B) The expression of miR-193a-3p, miR-210-3p and miR-5100 in normoxic or hypoxic BMSCs measured by Quantitative real-time PCR. Experiments were performed in triplicate.*P < 0.05. (TIF 216 kb)

Additional file 5: **Figure S5.** Exosomal miRNAs from BMSCs activate STAT3. (A) C57BL/6 mice were subcutaneously injected with LLC-RFP with or without BMSCs. The red fluorescent protein positive LLC cells were collected from the tumor sites by flow cytometry cell sorting and subjected to RNA sequencing analysis. Hierarchical clustering analysis was performed on differentially expressed mRNAs between LLC cells collected from mice that received co-injection of BMSCs and LLC or LLC injection alone. Columns represent individual samples and rows represent each gene. Red and green reflect high and low expression levels, respectively. (B) Cells that invaded to the bottom surface were stained with crystal violet and observed by light microscopy (magnification, 100×). The numbers of migrating cells or invading cells were counted from six fields of view in each group. Data were presented as the mean ± SD, and analyzed with Student’s t-test. *P < 0.05; ** < 0.01; *** < 0.001. (TIF 274 kb)

Additional file 6: **Figure S6.** Any combination of 2 exosomal miRNAs did not show higher specificity and sensitivity in discriminating patients. (A) ROC analysis of any combination of 2 exosomal miRNAs for cancer patients and non-cancerous controls. (B) ROC analysis of any combination of 2 exosomal miRNAs for the metastatic lung cancer patients and non-metastatic lung cancer patients. (TIF 837 kb)

## Abbreviations

BMSCs: Bone marrow-derived mesenchymal stem cells
DMEM: Dulbecco’s Modified Eagle’s medium
EMT: Epithelial mesenchymal transition
FBS: Fetal bovine serum
hBMSCs: Human bone marrow-derived mesenchymal stem cells
HIF: Hypoxia inducible factor
hnRNPs: Heterogeneous nuclear ribonucleoproteins
LLC: Lewis lung cancinoma cells
MEM: Minimum essential medium
miRNAs: microRNAs
NSCLC: Non-small cell lung cancer
NTA: Nanoparticle Tracking Analysis
OPSCC: Oropharyngeal squamous cell carcinoma
PBS: Phosphate Buffered Saline
PIAS3: Protein inhibitor of activated stat3
RBPs: RNA binding proteins
RCC: Renal cell carcinoma
ROC: Receiver operating characteristic
SOCS: Suppressor of cytokine signaling
STAT3: Signal transducers and activators of transcription 3
TEM: Transmission electron microscopy

## References

[1] Vallabhajosyula P, Korutla L, Habertheuer A, Yu M, Rostami S, Yuan CX (2017). Tissue-specific exosome biomarkers for noninvasively monitoring immunologic rejection of transplanted tissue. J Clin Invest.

[2] Zhang J, Li S, Li L, Li M, Guo C, Yao J (2015). Exosome and exosomal microRNA: trafficking, sorting, and function. Genomics Proteomics Bioinformatics.

[3] McDonald MK, Capasso KE, Ajit SK. Purification and microRNA profiling of exosomes derived from blood and culture media. J Vis Exp. 2013;(76):e50294. 10.3791/50294 PMC3727427 23792786

[4] Li XJ, Ren ZJ, Tang JH, Yu Q (2017). Exosomal MicroRNA MiR-1246 promotes cell proliferation, invasion and drug resistance by targeting CCNG2 in breast Cancer. Cell Physiol Biochem.

[5] Fang JH, Zhang ZJ, Shang LR, Luo YW, Lin YF, Yuan Y, et al. Hepatoma cell-secreted exosomal microRNA-103 increases vascular permeability and promotes metastasis by targeting junction proteins. Hepatology. 2018. 10.1002/hep.29920 29637568

[6] Cai Q, Zhu A, Gong L (2018). Exosomes of glioma cells deliver miR-148a to promote proliferation and metastasis of glioblastoma via targeting CADM1. Bull Cancer.

[7] Vanni I, Alama A, Grossi F, Dal Bello MG, Coco S (2017). Exosomes: a new horizon in lung cancer. Drug Discov Today.

[8] Zeng AQ, Yu Y, Yao YQ, Yang FF, Liao M, Song LJ (2018). Betulinic acid impairs metastasis and reduces immunosuppressive cells in breast cancer models. Oncotarget.

[9] Perez-Hernandez J, Olivares D, Forner MJ, Ortega A, Solaz E, Martinez F (2018). Urinary exosome miR-146a is a potential marker of albuminuria in essential hypertension. J Transl Med.

[10] Zhang X, Xin G, Sun D (2018). Serum exosomal miR-328, miR-575, miR-134 and miR-671-5p as potential biomarkers for the diagnosis of Kawasaki disease and the prediction of therapeutic outcomes of intravenous immunoglobulin therapy. Exp Ther Med.

[11] Kim JE, Eom JS, Kim WY, Jo EJ, Mok J, Lee K (2018). Diagnostic value of microRNAs derived from exosomes in bronchoalveolar lavage fluid of early-stage lung adenocarcinoma: a pilot study. Thorac Cancer.

[12] Villarroya-Beltri C, Baixauli F, Gutierrez-Vazquez C, Sanchez-Madrid F, Mittelbrunn M (2014). Sorting it out: regulation of exosome loading. Semin Cancer Biol.

[13] Wilson WR, Hay MP (2011). Targeting hypoxia in cancer therapy. Nat Rev Cancer.

[14] Pouyssegur J, Dayan F, Mazure NM (2006). Hypoxia signalling in cancer and approaches to enforce tumour regression. Nature.

[15] King HW, Michael MZ, Gleadle JM (2012). Hypoxic enhancement of exosome release by breast cancer cells. BMC Cancer.

[16] Eliasson P, Jonsson JI (2010). The hematopoietic stem cell niche: low in oxygen but a nice place to be. J Cell Physiol.

[17] Xu W, Zhang X, Qian H, Zhu W, Sun X, Hu J (2004). Mesenchymal stem cells from adult human bone marrow differentiate into a cardiomyocyte phenotype in vitro. Exp Biol Med (Maywood).

[18] Jiang C, Sun J, Dai Y, Cao P, Zhang L, Peng S (2015). HIF-1A and C/EBPs transcriptionally regulate adipogenic differentiation of bone marrow-derived MSCs in hypoxia. Stem Cell Res Ther.

[19] Kamerkar S, LeBleu VS, Sugimoto H, Yang S, Ruivo CF, Melo SA (2017). Exosomes facilitate therapeutic targeting of oncogenic KRAS in pancreatic cancer. Nature.

[20] Lasser C, Eldh M, Lotvall J. Isolation and characterization of RNA-containing exosomes. J Vis Exp. 2012;(59):e3037. 10.3791/3037 PMC3369768 22257828

[21] Wu H, Zhou J, Mei S, Wu D, Mu Z, Chen B (2017). Circulating exosomal microRNA-96 promotes cell proliferation, migration and drug resistance by targeting LMO7. J Cell Mol Med.

[22] Wang J, Yao Y, Wu J, Li G (2015). Identification and analysis of exosomes secreted from macrophages extracted by different methods. Int J Clin Exp Pathol.

[23] Fang JH, Zhang ZJ, Shang LR, Luo YW, Lin YF, Yuan Y (2018). Hepatoma cell-secreted exosomal microRNA-103 increases vascular permeability and promotes metastasis by targeting junction proteins. Hepatology.

[24] Qu Z, Wu J, Luo D, Jiang C, Ding Y (2016). Exosomes derived from HCC cells induce sorafenib resistance in hepatocellular carcinoma both in vivo and in vitro. J Exp Clin Cancer Res.

[25] Kroh EM, Parkin RK, Mitchell PS, Tewari M (2010). Analysis of circulating microRNA biomarkers in plasma and serum using quantitative reverse transcription-PCR (qRT-PCR). Methods.

[26] Arroyo JD, Chevillet JR, Kroh EM, Ruf IK, Pritchard CC, Gibson DF (2011). Argonaute2 complexes carry a population of circulating microRNAs independent of vesicles in human plasma. Proc Natl Acad Sci U S A.

[27] Chugh PE, Sin SH, Ozgur S, Henry DH, Menezes P, Griffith J (2013). Systemically circulating viral and tumor-derived microRNAs in KSHV-associated malignancies. PLoS Pathog.

[28] Khatua AK, Taylor HE, Hildreth JE, Popik W (2009). Exosomes packaging APOBEC3G confer human immunodeficiency virus resistance to recipient cells. J Virol.

[29] Zhang ZN, Xu JJ, Fu YJ, Liu J, Jiang YJ, Cui HL (2013). Transcriptomic analysis of peripheral blood mononuclear cells in rapid progressors in early HIV infection identifies a signature closely correlated with disease progression. Clin Chem.

[30] Alexander M, Hu R, Runtsch MC, Kagele DA, Mosbruger TL, Tolmachova T (2015). Exosome-delivered microRNAs modulate the inflammatory response to endotoxin. Nat Commun.

[31] Goldie BJ, Dun MD, Lin M, Smith ND, Verrills NM, Dayas CV (2014). Activity-associated miRNA are packaged in Map1b-enriched exosomes released from depolarized neurons. Nucleic Acids Res.

[32] Cogolludo A, Moreno L, Frazziano G, Moral-Sanz J, Menendez C, Castaneda J (2009). Activation of neutral sphingomyelinase is involved in acute hypoxic pulmonary vasoconstriction. Cardiovasc Res.

[33] Zhu J, Lu K, Zhang N, Zhao Y, Ma Q, Shen J, et al. Myocardial reparative functions of exosomes from mesenchymal stem cells are enhanced by hypoxia treatment of the cells via transferring microRNA-210 in an nSMase2-dependent way. Artif Cells Nanomed Biotechnol. 2017:1–12. 10.1080/21691401.2017.1388249 PMC5955787 29141446

[34] Hu X, Wu R, Shehadeh LA, Zhou Q, Jiang C, Huang X (2014). Severe hypoxia exerts parallel and cell-specific regulation of gene expression and alternative splicing in human mesenchymal stem cells. BMC Genomics.

[35] Bowler E, Porazinski S, Uzor S, Thibault P, Durand M, Lapointe E (2018). Hypoxia leads to significant changes in alternative splicing and elevated expression of CLK splice factor kinases in PC3 prostate cancer cells. BMC Cancer.

[36] Squadrito ML, Baer C, Burdet F, Maderna C, Gilfillan GD, Lyle R (2014). Endogenous RNAs modulate microRNA sorting to exosomes and transfer to acceptor cells. Cell Rep.

[37] Guduric-Fuchs J, O'Connor A, Camp B, O'Neill CL, Medina RJ, Simpson DA (2012). Selective extracellular vesicle-mediated export of an overlapping set of microRNAs from multiple cell types. BMC Genomics.

[38] Fan Q, Hu X, Zhang H, Wang S, You C, Zhang CY (2017). MiR-193a-3p is an important tumour suppressor in lung Cancer and directly targets KRAS. Cell Physiol Biochem.

[39] Mamoori A, Wahab R, Islam F, Lee K, Vider J, Lu CT (2018). Clinical and biological significance of miR-193a-3p targeted KRAS in colorectal cancer pathogenesis. Hum Pathol.

[40] Liu L, Li Y, Liu S, Duan Q, Chen L, Wu T (2017). Downregulation of miR-193a-3p inhibits cell growth and migration in renal cell carcinoma by targeting PTEN. Tumour Biol.

[41] Pan Y, Hu J, Ma J, Qi X, Zhou H, Miao X (2018). MiR-193a-3p and miR-224 mediate renal cell carcinoma progression by targeting alpha-2,3-sialyltransferase IV and the phosphatidylinositol 3 kinase/Akt pathway. Mol Carcinog.

[42] Lin M, Duan B, Hu J, Yu H, Sheng H, Gao H (2017). Decreased expression of miR-193a-3p is associated with poor prognosis in colorectal cancer. Oncol Lett.

[43] Wong N, Khwaja SS, Baker CM, Gay HA, Thorstad WL, Daly MD (2016). Prognostic microRNA signatures derived from the Cancer genome atlas for head and neck squamous cell carcinomas. Cancer Med.

[44] Block I, Burton M, Sorensen KP, Andersen L, Larsen MJ, Bak M (2018). Association of miR-548c-5p, miR-7-5p, miR-210-3p, miR-128-3p with recurrence in systemically untreated breast cancer. Oncotarget.

[45] Yang X, Shi L, Yi C, Yang Y, Chang L, Song D (2017). MiR-210-3p inhibits the tumor growth and metastasis of bladder cancer via targeting fibroblast growth factor receptor-like 1. Am J Cancer Res.

[46] Ren D, Yang Q, Dai Y, Guo W, Du H, Song L (2017). Oncogenic miR-210-3p promotes prostate cancer cell EMT and bone metastasis via NF-kappaB signaling pathway. Mol Cancer.

[47] Yan Y, Wang C, Zhou W, Shi Y, Guo P, Liu Y (2016). Elevation of circulating miR-210-3p in high-altitude hypoxic environment. Front Physiol.

[48] Petrozza V, Pastore AL, Palleschi G, Tito C, Porta N, Ricci S (2017). Secreted miR-210-3p as non-invasive biomarker in clear cell renal cell carcinoma. Oncotarget.

[49] Jorge K, Souza RP, Assis MTA, Araujo MG, Locati M, Jesus AMR (2017). Characterization of MicroRNA expression profiles and identification of potential biomarkers in leprosy. J Clin Microbiol.

[50] Mendell JT, Olson EN (2012). MicroRNAs in stress signaling and human disease. Cell.

[51] Zhang L, Li J, Wang Q, Meng G, Lv X, Zhou H (2017). The relationship between microRNAs and the STAT3-related signaling pathway in cancer. Tumour Biol.

