# Supplementary figures and images for: Hypoxic BMSC-derived exosomal miRNAs promote metastasis of lung cancer cells via STAT3-induced EMT
Source: Mol Cancer. 2019 Mar 13;18:40. doi: 10.1186/s12943-019-0959-5 (PMC6417285; doi:10.1186/s12943-019-0959-5)

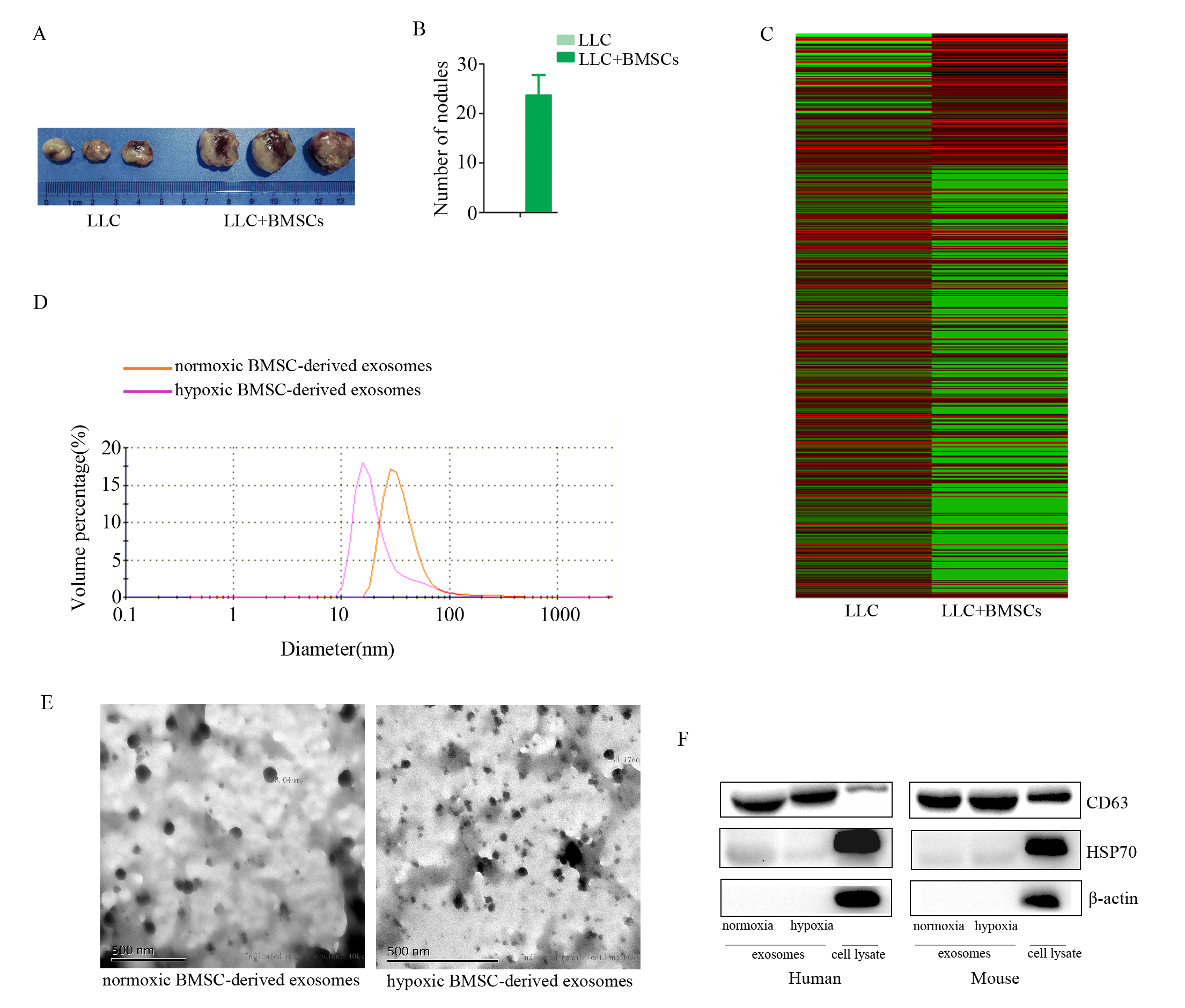

Supplement: Supplementary file 1 — Figure S1. Identifying exosomal miRNAs secreted from hypoxic BMSCs. LLC cells that were stably transfected with the firefly luciferase gene (luci-LLC) were subcutaneously injected with or without BMSCs into C57BL/6 mice. (A) The mice that received co-injection developed larger allograft tumours compared to the LLC cell injection alone. (B) Number of metastatic tumor nodules formed in the lungs was shown in histogram. (C) Hierarchical clustering analysis of plasma exosomal microRNA expression. Signals were normalized using Gene Spring GX software 11.0. Clustering was performed on differentially expressed exosomal microRNAs at FDR < 0.05. Columns represent individual samples and rows represent each exosomal microRNA. Red and green in cells reflect high and low expression levels, respectively, as indicated in the scale bar (log2-transformed scale). The BMSCs were cultured in a hypoxic chamber for 3 days. The exosomes were collected and isolated from the cell culture medium. (D) Size distribution analysis of purified exosomes by DLS (Zetasizer Nano ZS90 instrument, Malvern). (E) transmission electron microscopy images for exosomes(scale bar = 500 nm). (F) Exosomal markers (CD63,HSP70) were analyzed in exosomes and cell lysate by western blotting. β-actin was used as an internal reference. (TIF 1256 kb) [file 12943_2019_959_MOESM1_ESM.tif]

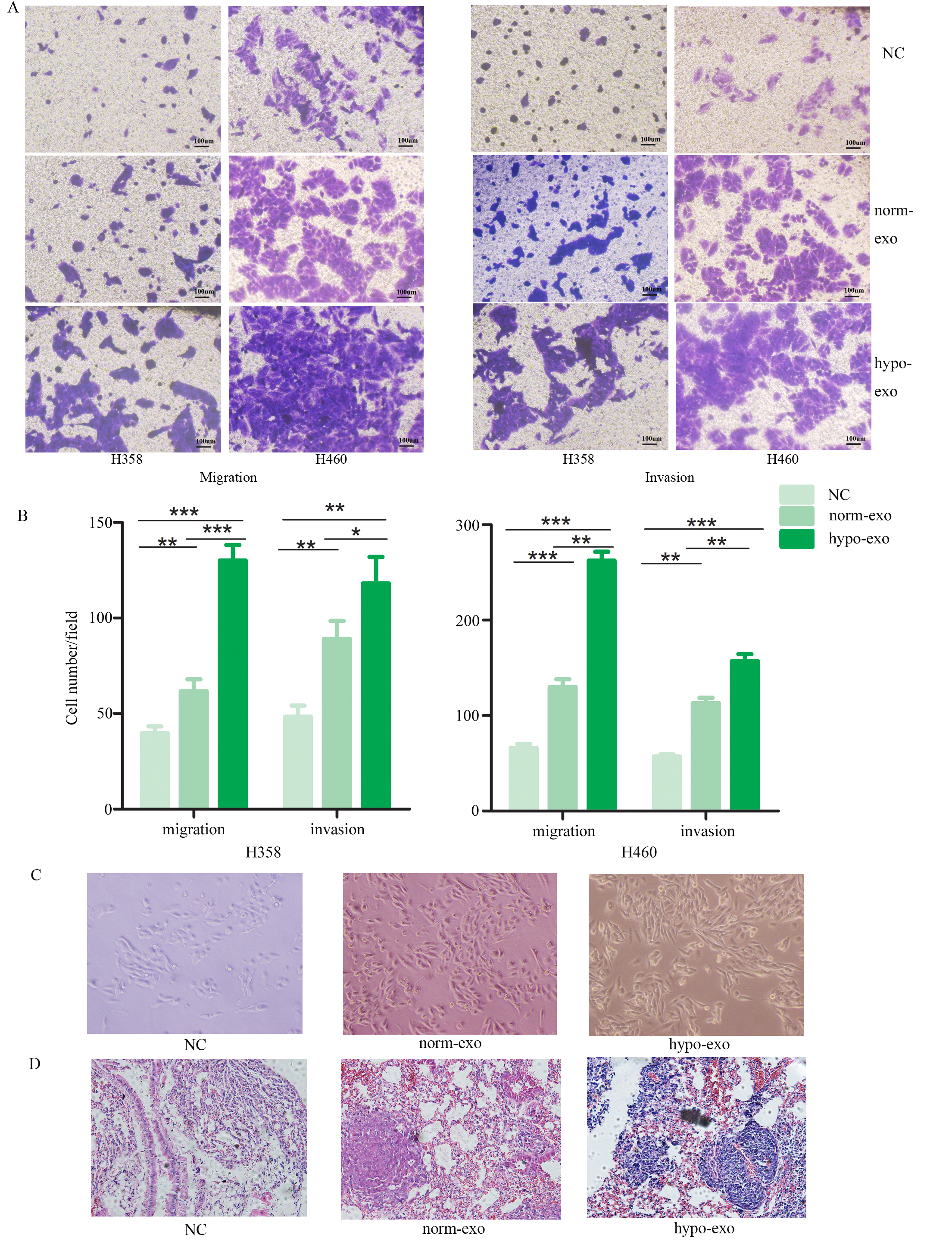

Supplement: Supplementary file 2 — Figure S2. Hypoxic BMSC-derived exosomes promote lung cancer cells migration and invasion. Cell migration and invasion were measured by transwell assays. (A) H358 and H460 Cells were treated with hypoxic BMSC-secreted or normoxic BMSC-secreted exosomes for 48 h. Cells that invaded to the bottom surface were stained with crystal violet and observed by light microscopy (magnification, 100×). (B) The numbers of migrating cells or invading cells were counted from six fields of view in each group. Data were presented as the mean ± SD, and analyzed with Student’s t-test. *P < 0.05; ** < 0.01; *** < 0.001. (C) Representative images of H358 after treatment with exosomes (magnification,100×). (D) Representative images of Hematoxylin and Eosin (HE) staining of lungs section to detect the metastatic nodules in the lung. (TIF 8240 kb) [file 12943_2019_959_MOESM2_ESM.tif]

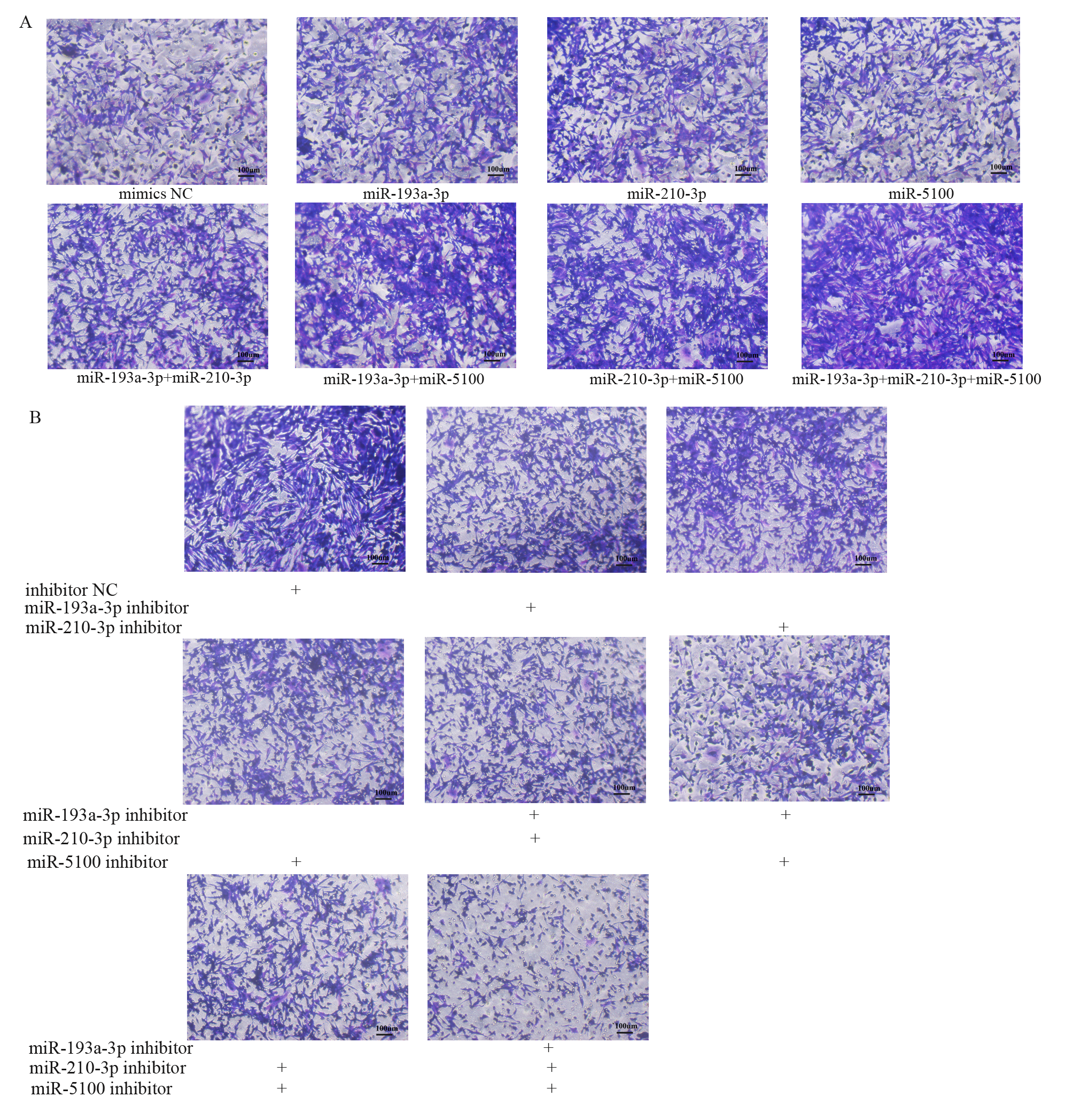

Supplement: Supplementary file 3 — Figure S3. Exosome-mediated transfer of miR-193a, miR-210-3p and miR-5100 from BMSCs promotes invasion of cancer cells. (A) Cell invasion were measured by transwell assays. Cells were transfected with mimics of miR-193a-3p, miR-210-3p and miR-5100. Cells that invaded to the bottom surface were stained with crystal violet and observed by light microscopy (magnification, 100×). (B) Cell invasion were measured by transwell assays. The invasion phenotype was reversed by select microRNA inhibitors. LLC cells were treated with hypoxic BMSC-secreted exosomes followed by transfection with inhibitors of miR-193a-3p, miR-210-3p and miR-5100. Cells that invaded to the bottom surface were stained with crystal violet and observed by light microscopy (magnification, 100×). (TIF 5905 kb) [file 12943_2019_959_MOESM3_ESM.tif]

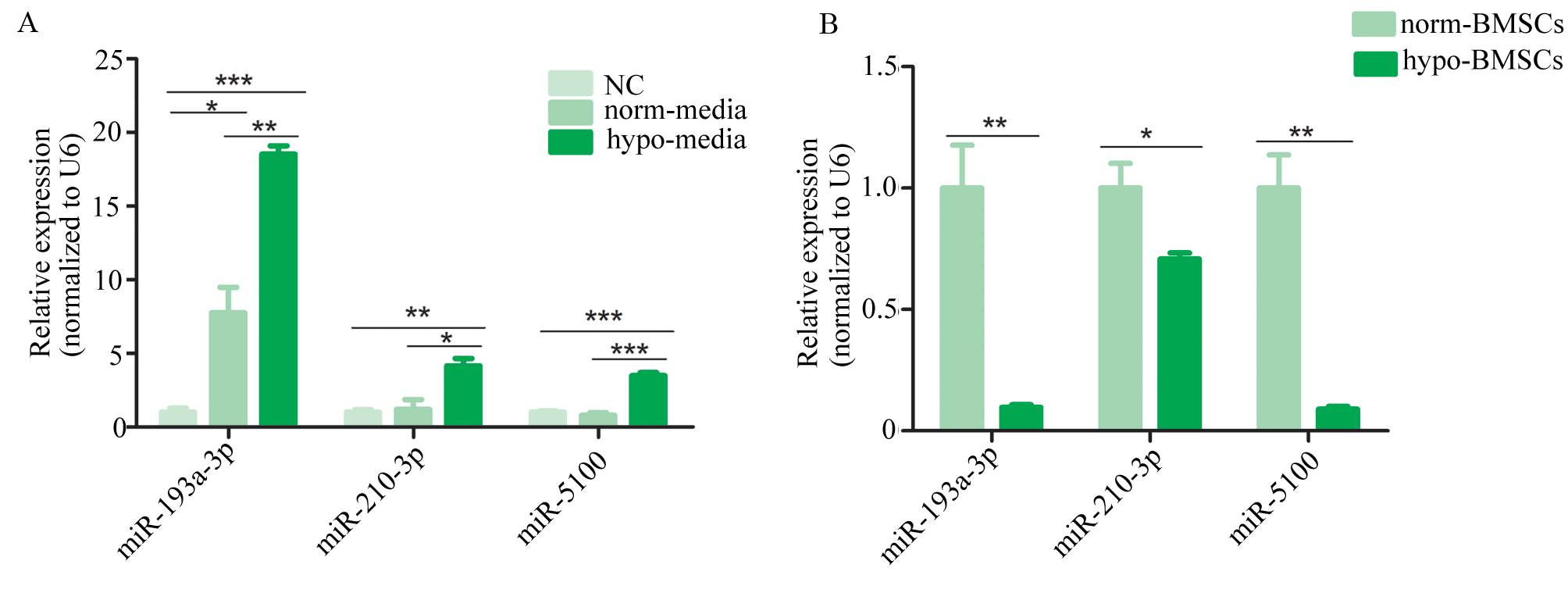

Supplement: Supplementary file 4 — Figure S4. Exosome-mediated transfer of miR-193a, miR-210-3p and miR-5100 from BMSCs to cancer cells. (A)The expression of miR-193a-3p, miR-210-3p and miR-5100 in cancer cells after treatment measured by Quantitative real-time PCR. LLC cells were treated with normoxic or hypoxic BMSC medium. Experiments were performed in triplicate. *P < 0.05; ** < 0.01; *** < 0.001. (B) The expression of miR-193a-3p, miR-210-3p and miR-5100 in normoxic or hypoxic BMSCs measured by Quantitative real-time PCR. Experiments were performed in triplicate.*P < 0.05. (TIF 216 kb) [file 12943_2019_959_MOESM4_ESM.tif]

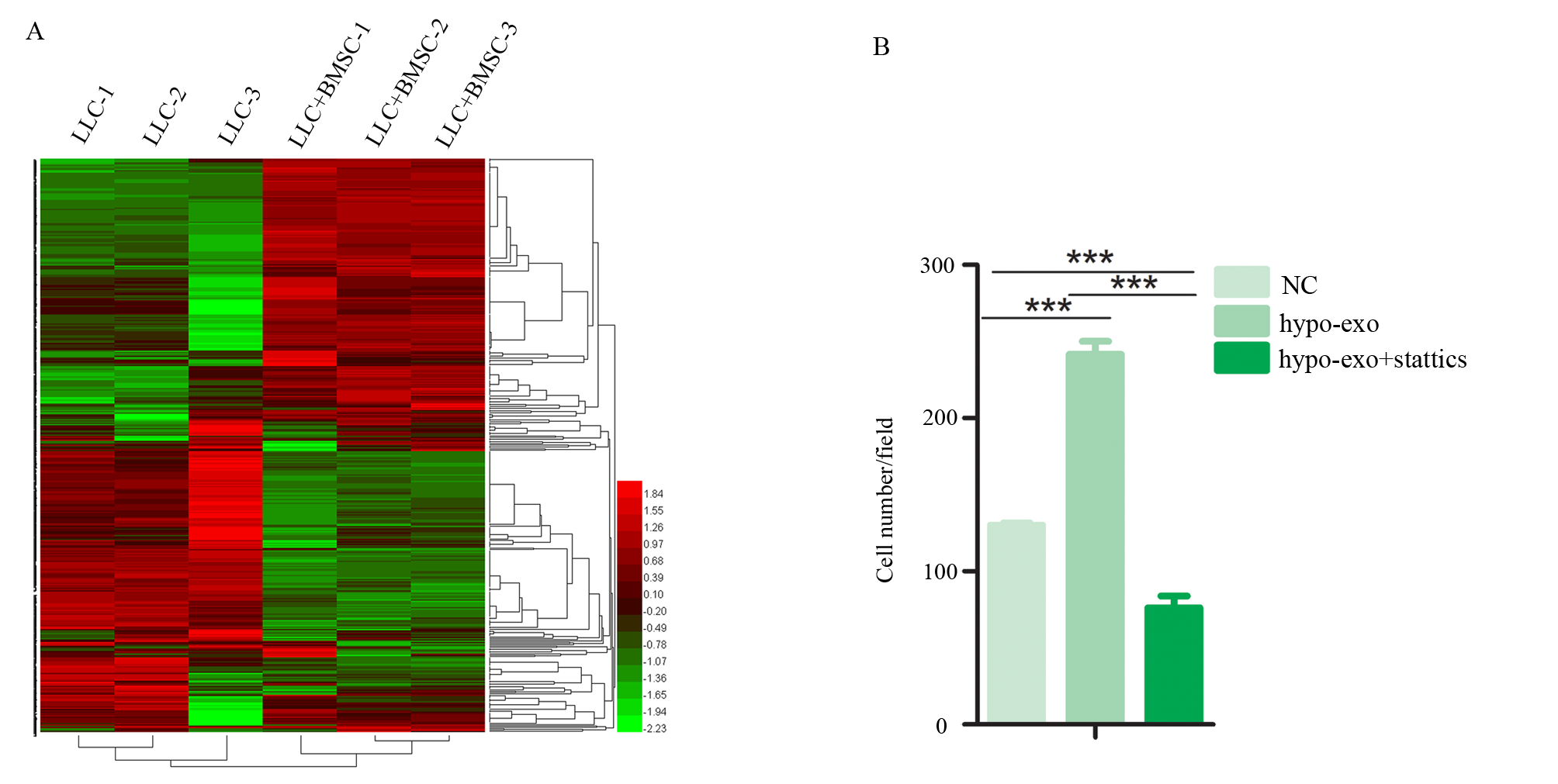

Supplement: Supplementary file 5 — Figure S5. Exosomal miRNAs from BMSCs activate STAT3. (A) C57BL/6 mice were subcutaneously injected with LLC-RFP with or without BMSCs. The red fluorescent protein positive LLC cells were collected from the tumor sites by flow cytometry cell sorting and subjected to RNA sequencing analysis. Hierarchical clustering analysis was performed on differentially expressed mRNAs between LLC cells collected from mice that received co-injection of BMSCs and LLC or LLC injection alone. Columns represent individual samples and rows represent each gene. Red and green reflect high and low expression levels, respectively. (B) Cells that invaded to the bottom surface were stained with crystal violet and observed by light microscopy (magnification, 100×). The numbers of migrating cells or invading cells were counted from six fields of view in each group. Data were presented as the mean ± SD, and analyzed with Student’s t-test. *P < 0.05; ** < 0.01; *** < 0.001. (TIF 274 kb) [file 12943_2019_959_MOESM5_ESM.tif]

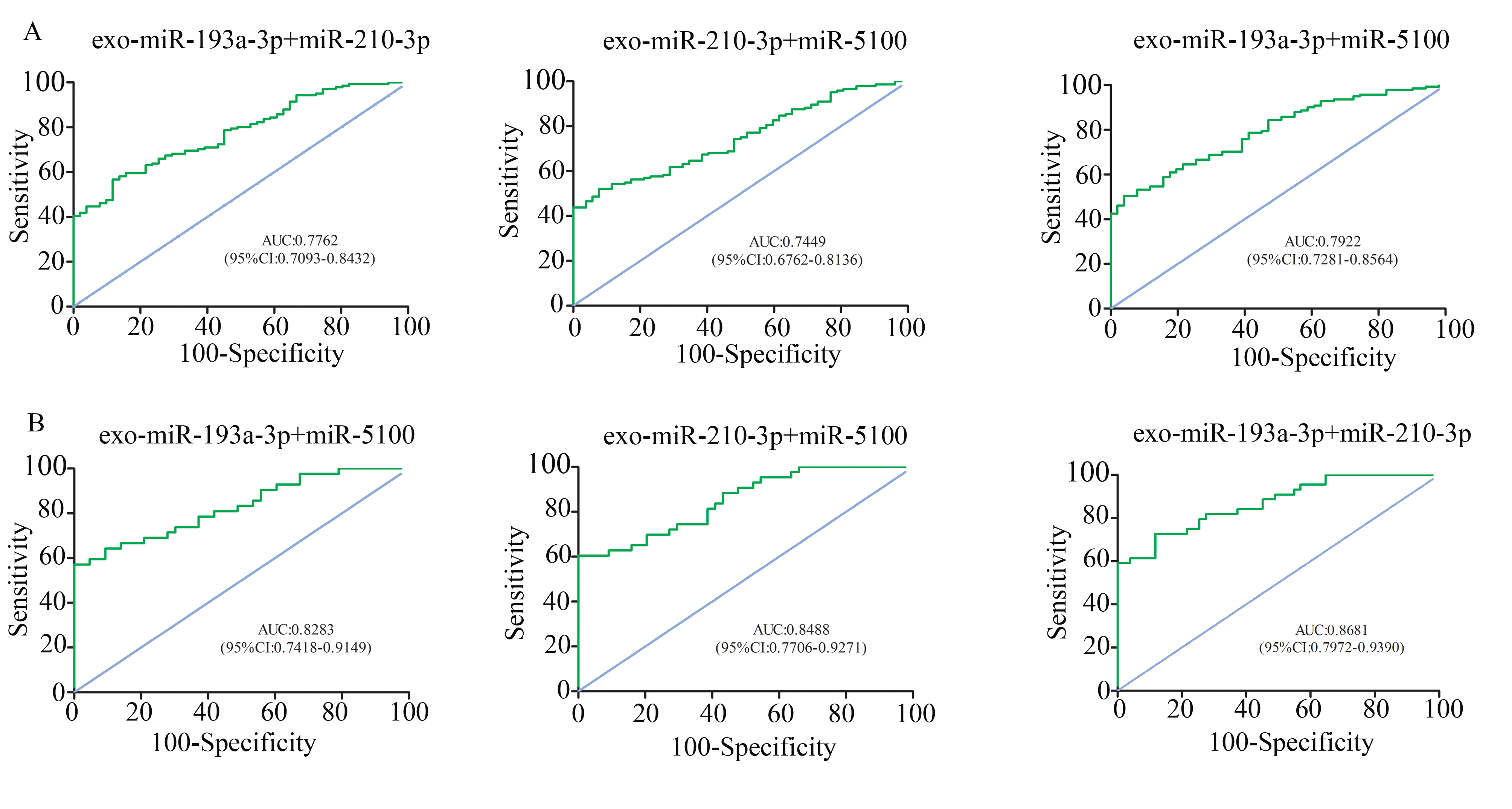

Supplement: Supplementary file 6 — Figure S6. Any combination of 2 exosomal miRNAs did not show higher specificity and sensitivity in discriminating patients. (A) ROC analysis of any combination of 2 exosomal miRNAs for cancer patients and non-cancerous controls. (B) ROC analysis of any combination of 2 exosomal miRNAs for the metastatic lung cancer patients and non-metastatic lung cancer patients. (TIF 837 kb) [file 12943_2019_959_MOESM6_ESM.tif]
